# Genetic Variation and Genome-Enabled Prediction of White Lupin Frost Resistance in Different Reference Populations

**DOI:** 10.3390/ijms262010224

**Published:** 2025-10-21

**Authors:** Nicolò Franguelli, Daniele Cavalli, Nelson Nazzicari, Luciano Pecetti, Tommaso Notario, Paolo Annicchiarico

**Affiliations:** Research Centre for Animal Production and Aquaculture, Council for Agricultural Research and Economics, 26900 Lodi, Italy; nicolo.franguelli@crea.gov.it (N.F.); daniele.cavalli@crea.gov.it (D.C.); nelson.nazzicari@crea.gov.it (N.N.); luciano.pecetti@crea.gov.it (L.P.); tommaso.notario@crea.gov.it (T.N.)

**Keywords:** abiotic stress, cold acclimation, frost tolerance, genetic architecture, genetic resources, genomic selection, GWAS, *Lupinus albus*, winter hardiness, winter mortality

## Abstract

In various European regions, white lupin (*Lupinus albus* L) production could increase by autumn sowing of winter-hardy varieties. This study aimed to explore the genetic variation, the genetic architecture, and the genomic prediction of frost resistance in two reference populations, one including 144 landrace and cultivar genotypes, and the other comprising 144 breeding lines. These populations were genotyped by 40,914 and 32,951 SNP markers, respectively, issued by genotyping-by-sequencing. The genotypes were phenotyped for mortality and a biomass injury score at freezing temperature of −11 °C under controlled conditions. Both traits, highly correlated, exhibited large genetic variation and high broad-sense heritability (*H*^2^ = 0.76–0.82). A genome-wide association study highlighted their polygenic architecture and detected markers linked to candidate genes. The intra-population predictive ability of plant mortality achieved 0.41 for landrace and cultivar germplasm, and 0.67 for breeding lines. The cross-population predictive ability was higher when using the model constructed for landrace and cultivar germplasm to predict breeding lines (0.39) than the reverse (0.26). Landrace field survival was largely influenced by late phenology in addition to frost resistance. Our results revealed frost-resistant germplasm, confirmed the polygenic control of frost resistance, and highlighted genomic prediction opportunities for line selection and the identification of elite genetic resources.

## 1. Introduction

White lupin (*Lupinus albus* L.) is a minor crop of increasing interest for European agriculture. As a rainfed cool-season grain legume, it can counteract soil fertility loss, enhance farming sustainability, and reduce water use [1]. In addition, wider grain legume cultivation can reduce the European dependence on international markets for high-protein feedstuffs and their associated environmental costs [2,3]. Because of its outstanding seed protein content (nearly 40% on dry matter [4]), white lupin showed higher crude protein yield per unit area than other grain legumes such as pea (*Pisum sativum* L.), faba bean (*Vicia faba* L.) and narrow-leafed lupin (*Lupinus angustifolius* L.) in southern Europe [5]. Such a high protein content is complemented by other favorable characteristics, namely, a high level of essential amino acids, valuable techno-functional properties, positive effects on human health with respect to diabetes, hypertension, cardiovascular diseases, and obesity [6], and a content of 8–12% of oil with excellent nutritional characteristics [7]. Therefore, white lupin could meet the growing demand of pulses for novel food products [8,9]. Nevertheless, higher crop yields are needed for this and other grain legumes to increase the economic sustainability of their cultivation in Europe [10].

Frost and terminal drought are major factors limiting the yield of autumn-sown cool-season grain legumes in Europe. Winter hardiness (as estimated by winter plant survival) is essentially determined by the effect of frost [11], although other factors (such as waterlogging or fungal diseases) may affect it. Spring sowing is currently adopted in cold-prone regions of Europe to avoid frost events, but the changing climate is expected to promote the expansion of autumn sowing northward, allowing for potentially higher crop yields via a longer crop cycle and terminal drought escape due to earlier harvesting [12]. Even in Mediterranean environments, frost remains a major constraint because its damaging effects may be enhanced by poor plant acclimatation caused by mild winters [13]. The changing climate is expected to increase the occurrence of mild winters on the one hand, and the risk of sudden, early or late frost events on the other [14]. Late flowering by high vernalization requirement, which allows the sensitive reproductive organs under development to escape frost [15,16], and frost resistance are the key components of winter hardiness. A late phenology, however, involves greater susceptibility to terminal drought [17,18]. Therefore, white lupin breeding for autumn sowing in drought-prone regions should aim to combine the phenology-unrelated resistances to frost on the one hand and to terminal drought on the other in material with an intermediate phenology. Frost resistance, which could be synthetically expressed as a positive deviation from the genotype winter mortality value expected according to its onset of flowering [19], relies on physiological modifications that prevent or resist intracellular ice formation [20,21]. Such modifications may be activated by daylength reduction [22,23], and by exposure to low, non-freezing temperatures in a process known as cold acclimation or hardening [24,25,26].

There is limited knowledge on the extent of genetic variation for frost resistance in white lupin. Germplasm pools of landraces from the Azores, Italy, Greece, and Madeira and the Canary Islands have been reported as valuable genetic resources for winter hardiness observed in field experiments [15,16,27]. However, field-based winter survival depends on various factors, and its assessment is complicated by the wide and increasing climatic variation across years (which reduces the applicability and replicability of the results) [28]. Controlled environments, where hardening conditions and frost intensity and duration can be standardized, can overcome the limitations of field trials and allow for a rapid and possibly off-season evaluation of genotype responses [29]. Plant evaluation at an early growth stage can rule out any effect of flowering time and focus, therefore, on frost resistance. A previous study developed a protocol for large-scale evaluation of white lupin frost resistance in a walk-in growth chamber, identifying −11 °C as the optimal freezing temperature [19]. That study showed good consistency between genotype responses in this phenotyping platform (relative to plant mortality and a visual score of plant injury) and field-based winter mortality of the genotypes.

Large-scale germplasm evaluations under controlled conditions can be used for identifying elite genetic resources, selecting breeding material, and/or defining marker-assisted or genome-enabled procedures which could be used for future selection or identification of useful genetic resources in germplasm collections. The definition of such procedures achieved remarkable advances through the advent of next-generation sequencing techniques, such as genotyping-by-sequencing (GBS) [30]. GBS proved to be capable of generating thousands of polymorphic single-nucleotide polymorphism (SNP) markers for white lupin genetic analyses [31], and was used to define genomic prediction models for white lupin yielding ability in contrasting environments [32], resistance to drought [18], adaptation to calcareous soils [33], and resistance to anthracnose [34,35]. An issue of remarkable practical interest for genomic prediction models is the predictive ability of a model developed for a genetically broad reference population, such as an international germplasm collection, when applied onto a genetically narrower reference population, such as a collection of breeding lines [36]. For oligogenic traits, the possibility of identifying candidate genes through a genome-wide association study (GWAS) has been enhanced by the publication of a high-quality white lupin genome [37] on which GBS-generated markers can be aligned. Markers associated with frost resistance have already been identified in various legumes, such as faba bean [38], pea [39,40], and red clover (*Trifolium pratense* L.) [41].

This study focused on two white lupin reference populations, one including a world collection of landrace and cultivar genotypes (reference population 1) and the other comprising breeding lines issued from crosses of elite modern (sweet-seed) germplasm with landrace material (reference population 2). These populations were evaluated for frost resistance by the method described in [19]. The study aimed at assessing (a) the genetic variation for frost resistance in each population; (b) the relationship between controlled and field conditions for plant mortality; (c) the genetic architecture of the frost resistance trait, and putative candidate genes, based on GWAS analyses; (d) the predictive ability of genome-enabled models developed for each reference population; and (e) the cross-population predictive ability of the genomic prediction models, particularly with respect to the model developed for landrace and cultivar material when used for the prediction of breeding lines.

## 2. Results

### 2.1. Frost Resistance Variation and Relationship with Field-Based Winter Mortality

**Figure 1:**
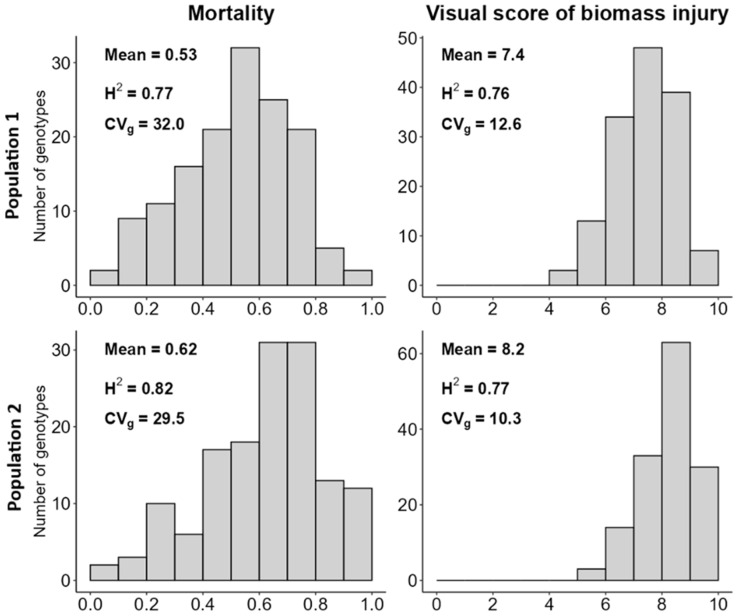
Distribution, mean, broad-sense heritability on an entry mean basis (*H*^2^), and genetic coefficient of variation (*CV_g_*) for proportion of plant mortality and visual score of biomass injury of landrace and cultivar genotypes (reference population 1) and breeding lines (reference population 2) of white lupin assessed at −11 °C freezing temperature in a phenotyping platform.

The frost resistance of the two reference populations was assessed in terms of plant mortality and a visual score of biomass injury following a freezing temperature of −11 °C applied to previously hardened plants. The genotype variation for these traits was wide (Figure 1) and statistically significant at *p* < 0.001 for both populations. The proportion of plant mortality ranged from 0.08 to 0.96 with a mean value of 0.53 for reference population 1 (landrace and cultivar genotypes), and from 0.05 to 1.00 with a mean value of 0.62 for reference population 2 (breeding lines). On average, population 2 tended to be more susceptible to frost than population 1 according to mean values of the biomass injury score (8.2 vs. 7.4). The values of plant mortality and biomass injury score of the individual genotypes are reported in Table S1 for landrace and cultivar germplasm and Table S2 for breeding lines. The top-ranking germplasm for plant survival was a genotype extracted from the Greek landrace Gr56 and the French winter-type cultivar Ludet (with mortality ≤ 0.09).

Broad-sense heritability values (*H*^2^) were high for both traits, ranging from 0.76 to 0.82 (Figure 1). Plant mortality and the visual score of biomass injury provided highly consistent information on genotype frost resistance, as indicated by their strong correlations (*r* = 0.97 and 0.96 for reference populations 1 and 2, respectively; *p* < 0.001). Plant mortality tended to be more effective than the injury score in capturing the genetic variation for frost resistance according to its wider dispersal for genotype frequency data and its higher genetic coefficient of variation (*CV_g_*) in Figure 1. Each of these traits exhibited somewhat higher *CV_g_* values for reference population 1 relative to population 2, in agreement with the expectation of greater variation in a world collection of landrace and cultivar material than in a collection of inbred lines derived from a fairly restricted parent group.

Landrace and cultivar genotypes of reference population 1 were grouped into 14 germplasm pools. Landraces were classified into 11 pools according to their geographical origin, while cultivars were classified into winter, spring, and Mediterranean phenological types. An analysis of variance revealed significant differences (*p* < 0.001) between pools for mean values of plant mortality and biomass injury score. On average, winter-type cultivars were highly frost-resistant according to mortality and biomass injury score values, followed by Mediterranean-type cultivars (Table 1). The winter-type cultivar pool was more resistant than any landrace pool, whereas the pool of spring-type cultivars was highly susceptible (Table 1). However, few differences between germplasm pools were statistically significant, owing to the large variation within germplasm pools (as highlighted by the range of values in Table 1). The greatest difference emerging among landrace pools was the high average frost susceptibility of material from Maghreb, which contrasted with the moderately high resistance exhibited by landraces from Greece, Spain, and the Atlantic islands (Table 1).

Four landraces from reference population 1 had been used as parents for the development of the breeding lines (reference population 2). We found consistency between the landrace parent value and the mean value of their progenies for plant mortality and visual injury score. In particular, the parent landrace with the greatest resistance (the Greek accession Gr56) produced breeding lines with the highest resistance, whereas the parent landrace with the greatest frost susceptibility (LAP123) generated lines with the highest susceptibility (Table 2).

**Figure 2:**
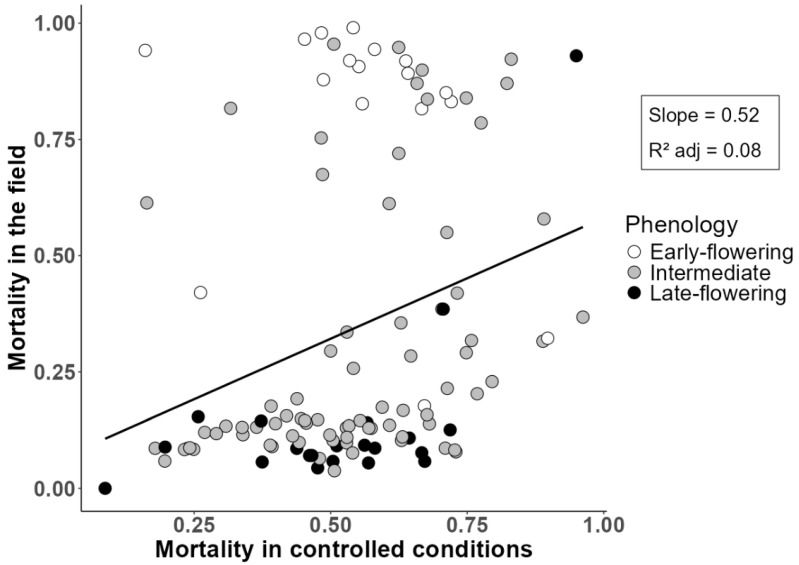
Linear regression of the proportion of plant mortality under field conditions as a function of the plant mortality in a phenotyping platform for 115 landraces and cultivars. Accessions are classified into three phenological classes based on the onset of flowering (class range values of flowering in days from January 1 are 94.0–115.0 for early flowering, 115.1–120.6 for intermediate flowering, and 120.7–130.7 for late flowering).

A total of 115 landraces and cultivars from reference population 1 had previously been evaluated under field conditions for winter plant mortality and onset of flowering [15]. The correlation between plant mortality under field conditions (as determined by stress resistance and stress escape by a late phenology) and plant mortality in the phenotyping platform (as determined essentially by frost resistance) was fairly low albeit significant (*r* = 0.29; *p* < 0.01). A linear regression of field-based plant mortality as a function of platform-based mortality with an indication of three phenological groups of accessions (early, intermediate, and late flowering) highlighted the role of phenology in determining the field mortality (Figure 2). Many accessions showing greater field mortality than the expected value based on platform mortality featured early flowering; whereas many accessions displaying lower field mortality than the platform-based expectation were characterized by late flowering (hence, greater phenology-based stress escape).

### 2.2. Analysis of Linkage Disequilibrium Decay and Population Structure, and Genome-Wide Association Study

The molecular characterization was based on 40,914 polymorphic SNPs for landrace and cultivar material (reference population 1), and 32,951 SNPs for breeding lines (reference population 2). The linkage disequilibrium (LD) decay plots for white lupin chromosomes based on Pearson’s correlation (*r*^2^) against physical distance are reported in Figures S1 and S2, respectively, for these populations. The LD decay threshold (i.e., the critical *r*^2^ value above which LD is likely attributable to physical linkage) was computed for each population as the 95th percentile of the distribution of pairwise inter-chromosomal *r*^2^ estimates after a square root transformation. The threshold was 0.046 for reference population 1, and 0.067 for population 2. For each chromosome within each population, LD extent values are reported in Table S3.

The population structure was investigated by discriminant principal components analysis (DPCA) on genotype data previously pruned for excess LD. For landrace and cultivar material, the DPCA identified one major discriminant function that classified the genotypes into two broad groups (Figure S3). Group 1 included the entire germplasm pools from Azores and Greece, whereas group 2 comprised the pools from Italy, the Maghreb, and winter-type cultivars, along with most of the genotypes from Egypt, Madeira and the Canaries, West Asia, and spring-type cultivars. All the remaining pools were distributed almost equally between the two groups. The DPCA for reference population 2 grouped the genotypes mainly on the ground of the 16 crosses they derived from (Figure S4). One discriminant function was retained for reference population 1 and 13 were retained for reference population 2 according to the a-score criterion, for use as covariates in the GWASs to compensate for population structure.

**Figure 3:**
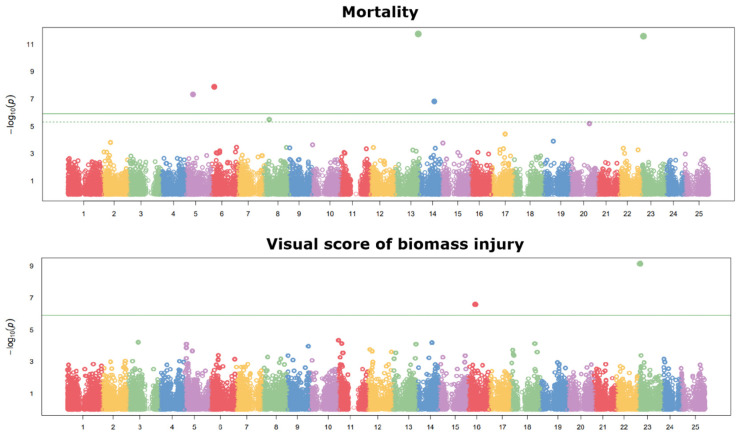
Manhattan plots showing the association scores of 40,914 SNPs (ordered along chromosomes) with the proportion of plant mortality and a visual score of biomass injury assessed at −11 °C freezing temperature in a phenotyping platform for 144 white lupin landrace and cultivar genotypes (reference population 1). The green continuous line is the Bonferroni threshold at 5%, whereas the green dashed line (where visible) is the false discovery rate threshold at 5%.

**Figure 4:**
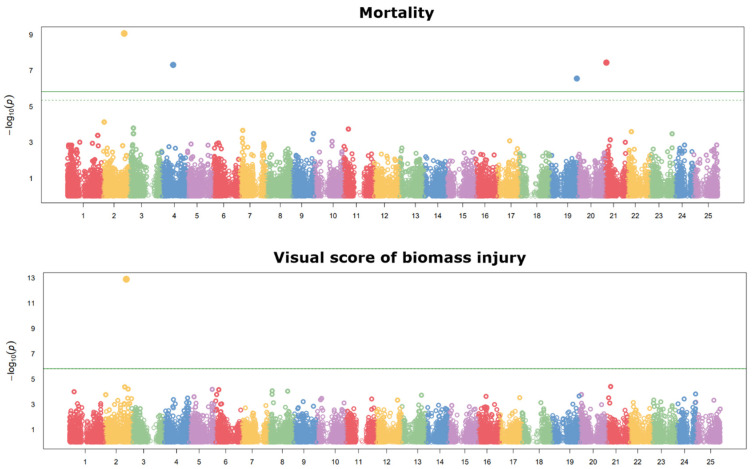
Manhattan plots showing the association scores of 32,951 SNPs (ordered along chromosomes) with the proportion of plant mortality and a visual score of biomass injury assessed at −11 °C freezing temperature in a phenotyping platform for 144 white lupin breeding lines (reference population 2). The green continuous line is the Bonferroni threshold at 5%, whereas the green dashed line (where visible) is the false discovery rate threshold at 5%.

The results of the GWASs are summarized in the Manhattan plots reported in Figure 3 for landrace and cultivar material and in Figure 4 for breeding lines. Significant SNPs were selected according to the false discovery rate (FDR) threshold at 5%. For reference population 1, five SNPs (on chromosomes 5, 6, 8, 13, and 14) were associated with plant mortality, one SNP (on chromosome 16) was associated with the visual score of biomass injury, and one SNP (on chromosome 23) was associated with both traits. For reference population 2, three SNPs (on chromosomes 4, 19, and 21) were associated with plant mortality, and one SNP (on chromosome 2) was associated with both traits. The list of significant SNPs and their association scores, minor allele frequency, and estimated trait effects is provided in Table S4. On the whole, the inconsistency for significant SNPs across populations, the presence of several significant SNPs in population 1, and the presence of several minor peaks of SNP association not reaching significance in both populations (Figure 3 and Figure 4) indicated a polygenic genetic architecture of frost resistance-related traits.

Out of 11 significant SNPs in the GWASs, 10 were found to be associated with at least one candidate gene on the white lupin genome browser (www.whitelupin.fr), by searching within genomic regions corresponding to the chromosome-specific LD extent in both directions. Only the significant SNP on chromosome 23 (associated with both traits in population 1) was not associated with any candidate gene, suggesting either a false positive or, more probably, the presence of a regulatory region affecting the transcription of a relevant gene. The full list of candidate genes, with putative encoded proteins and their putative roles in frost resistance, is provided in Table 3.

### 2.3. Genomic Selection

The predictive ability of genomic selection was assessed with respect to plant mortality and the visual biomass injury score by three statistical models: rrBLUP, Bayesian Lasso, and BayesB. We envisaged an intra-population scenario by building and testing models on the same population (using 10-fold cross validations), as well as a cross-population scenario by constructing models on one population and testing their predictive ability on the other population. The three models usually performed similarly. However, Bayesian Lasso provided a slightly higher predictive ability in most cases, while rrBLUP outperformed the other models for both traits for intra-population predictions of reference population 1. Predictive ability values of the best-performing models for each trait, reference population, and prediction scenario are reported in Table 4. On average, the predictive abilities did not differ substantially between the two traits. For both traits, intra-population predictive abilities were high for the population of breeding lines (0.672–0.678) and moderately high for that of landrace and cultivar material (0.414–0.376). Remarkably, the model constructed from landrace and cultivar data exhibited a minimal or nil drop in predictive ability passing from intra-population prediction for the same population to cross-population prediction for the population of breeding lines (0.414 vs. 0.393, for plant mortality; 0.376 vs. 0.386, for the visual injury score; Table 4), indicating its broad applicability. In contrast, the model constructed for the reference population featuring smaller genetic diversity, i.e., the breeding line population, displayed a drastic decrease in predictive ability passing from intra-population prediction to cross-population prediction applied to the genetically broader reference population of landraces and cultivars (0.672 vs. 0.255, for plant mortality; 0.678 vs. 0.232, for the visual injury score; Table 4). However, the transferability to the population of breeding lines of the prediction model constructed for landrace and cultivar material involved a decrease in predictive ability in the range of 41–43% relative to the model specifically developed for the breeding lines (0.393 vs. 0.672, for plant mortality; 0.386 vs. 0.678, for the visual injury score; Table 4).

## 3. Discussion

This study provided the first large-scale assessment of white lupin genotype frost resistance under controlled conditions, capitalizing on earlier work that optimized the evaluation procedure in these conditions [19]. The assessment was based on two traits, namely, plant mortality and the visual injury score, which were highly correlated. The former trait tended to be more sensitive than the latter in detecting relevant genetic variation in the current experiment layout (which included 15 plants per experimental unit and 144 evaluated genotypes per cycle). However, the visual injury score may be preferable in the presence of fewer plants per experimental unit (as envisageable when evaluating more genotypes per growth cycle), owing to greater experiment error expected for plant mortality in that case. A similar visual injury score was adopted when testing four plants per experimental unit in pea [40] and faba bean [25].

As expected, the genetically broader reference population 1 (including an international set of landrace and cultivar genotypes) exhibited larger variation for frost resistance than the reference population of breeding lines. The average proportion of plant mortality around 0.5 displayed by the former population was close to the average mortality displayed at the same freezing temperature by a smaller set of genotypes used to identify −11 °C as the optimal temperature for frost resistance evaluation [19]. Results for the individual genotypes (summarized in Tables S1 and S2) can help identify genetic resources with outstanding frost resistance for use as parent germplasm in breeding programs. Actually, the highly frost-resistant landrace Gr56 was already used as one of the parents that generated the breeding lines of reference population 2. The substantial consistency between parent and progeny values that we observed for frost resistance traits is comforting for breeding and agrees with a similar result observed for key seed quality traits such as protein content, total content of quinolizidine alkaloids, and seed weight [42]. In addition, the selection for higher frost resistance under controlled conditions could be favored by high broad-sense heritability and substantial genetic variation according to the present results.

Winter-type and Mediterranean-type cultivars ranked in this order for frost resistance and tended to display greater resistance than any landrace germplasm pool. This result reflected the effort on cold tolerance improvement performed by a few breeding programs, especially in France, where autumn sowing was devised as a key strategy for crop yield improvement [27]. In addition, frost resistance may be important even in the Mediterranean climate [13,29]. Landrace material from Greece, the Atlantic islands (Azores, Madeira and the Canary Islands) and Italy was previously reported as winter-hardy under field conditions [15,16,27]. While tending to confirm these findings in terms of frost resistance, our results indicated relatively modest differences between most germplasm pools for this characteristic, suggesting that frost escape by a late phenology has a key impact on field-based winter hardiness. Indeed, a multi-location evaluation of landrace germplasm pools highlighted the close relationship between a late phenology and the specific adaptation to a cold-prone environment of northern Italy of germplasm from the Azores and Greece [15]. The modest correlation currently observed for plant mortality across phenotyping platform and field conditions (*r* = 0.29), and the importance of flowering time in accounting for plant mortality differences across these conditions (Figure 2), reinforced this finding, suggesting that phenology had a greater impact than frost resistance on the field-based winter survival of landrace accessions. Phenology emerged as a major determinant of the specific adaptation to cold-prone or mild-winter Italian environments also for other cultivar and breeding line materials [15]. However, the modest correlation observed for plant mortality across platform and field conditions was emphasized by the wide variation for onset of flowering expressed by the evaluated material [15], and a higher correlation would occur for a material with a similar phenology. Delayed flowering is mediated by vernalization requirements and the accumulation of growing degree days [43,44,45]. Studies on other legumes reported higher correlations for plant mortality across artificial and field conditions, which approached 0.7 [46] and 0.5–0.6 for pea [47], and 0.5 for faba bean [48] and red clover [49]. As a matter of fact, a lack of correlation across these conditions may result not only because of the importance of phenology-based stress escape but also because of other factors such as waterlogging and fungal diseases, particularly *Pleiochaeta setosa*, *Fusarium* spp., and *Colletotrichum gloeosporioides* (anthracnose) [16,50]. As anticipated, our focus on selection for frost resistance (as allowed by the evaluation of young plants in our phenotyping platform) has particular interest for southern Europe and other regions that are prone to both frost and terminal drought, since selection for a late phenology would lead to material with high winter hardiness but high susceptibility to terminal drought.

The genetic improvement of white lupin frost resistance may rely not only on phenotypic selection under controlled conditions (which ideally requires a high-throughput phenotyping platform) but also on marker-assisted or genomic selection. The results of the DPCA that preceded the GWAS showed a weak population structure for reference population 1, with just a loose relationship with the geographic origin of landraces that confirmed earlier results [34,51]. The two major genetic groups identified in this population were not clearly related to frost resistance of the material, since frost-resistant, winter-type cultivars and frost-resistant landrace pools (such as those from Greece and the Azores) clustered in different groups. As expected, the population structure of reference population 2 reflected the crosses from which the lines originated. The slower average LD decay in reference population 2 compared to population 1 was consistent with the lower number of meiotic events that occurred in the breeding line population compared to the population of landrace and cultivar genotypes. Overall, LD decay values confirmed earlier observations [51] of rapid LD decay in white lupin relative to other inbred crops, which complicates the identification of significant SNPs in this species.

The GWAS identified various population-specific SNPs associated with plant mortality and/or the biomass injury score. On the whole, it indicated a polygenic genetic control of frost resistance that agrees with the complexity of frost resistance mechanisms. These mechanisms are activated by exposure to low, non-freezing temperatures (during cold acclimation or hardening), and enhance plant survival during later freezing events by preventing intracellular ice formation or resisting ice damage. This occurs through a decrease in shoot water content [21], an increase in cell membrane stability by changing the lipid-to-protein ratio and the membrane lipid unsaturation level [25], and an accumulation of cryoprotectant and osmoprotectant compounds that protect against dehydration [26,52]. Various of the current candidate genes listed in Table 3 appear to be involved in the cold signaling pathway, acting as primary sensors, signal modulators, or regulators of gene expression. In particular, Chr16g0384801 and Chr14g0368501 encode, respectively, a putative receptor-like kinase (RLK) and a leucine-rich repeat (LRR) domain protein, both of which are membrane-localized and can act as primary sensors when low temperatures are perceived through changes in membrane fluidity [53]. Chr21g0306351 and Chr02g0156401 encode, respectively, a putative serine/threonine phosphatase and a metallo-dependent phosphatase. Phosphatases are known to negatively modulate key signaling proteins involved in stress responses. In alfalfa (*Medicago sativa* L.), the activity of serine/threonine phosphatase type 2A (PP2A) is inhibited by cold, and its inactivation results in increased kinase activity and transmission of cold signals to the nucleus [54]. Chr08g0234251 encodes a chromatin regulator of the PHD family. In rye, ScPHD5, belonging to the plant homeodomain (PHD) family, regulates C-repeat binding factor (CBF) gene expression, one of the most important transcription factors in the response to low temperatures [55]. Three other candidate genes are likely to be involved in protection from intra-cellular ice formation. Chr04g0256531 encodes a putative hydrolase and, in *Arabidopsis*, two hydrolase enzymes, namely XTH19 and XTH22, strengthen the cell wall during cold and sub-zero acclimation, making cells more resistant to freezing mechanical stress [56,57]. Chr13g0303301 and Chr04g0256541 encode, respectively, a putative transcription factor of the bHLH family (which is associated with enhanced proline accumulation [58]) and a putative potassium transporter (which regulates the uptake of potassium ions (K^+^) in plant cell membranes [59]). Potassium ions and proline are among the major cryoprotectants and osmoprotectants, which lower the freezing point of cellular fluids, inhibit ice formation, and at the same time help maintain cell osmolarity and prevent severe dehydration caused by membrane damage due to ice formation [52]. Indeed, 11 genes encoding members of the bHLH protein family were detected as candidate genes for frost resistance in alfalfa [60]. Another negative effect of abiotic stress on cell metabolism is the overproduction of reactive oxygen species (ROS), which damage molecular and cellular components due to the oxidation of biomolecules such as lipids, carbohydrates, proteins, enzymes, and DNA [61]. Chr06g0163651 encodes a putative oxidoreductase that can enhance ROS scavenging. Accumulation of oxidoreductase enzymes during cold acclimation was observed in *Solanum tuberosum*, *Festuca pratensis*, and *Arabidopsis* [62,63,64]. Moreover, Chr13g0303311 encodes putative ribosomal protein S4/S9. Ribosomal proteins are crucial for ribosomal biogenesis, which allows the rapid synthesis of proteins involved in cold stress response, and various studies suggest the contribution of ribosome-related genes to frost resistance [63,65]. Finally, Chr19g0139891 and Chr05g0219341 encode, respectively, a putative vacuolar protein sorting-associated protein, and a putative CBS domain-containing protein (CDCP), which appear to be associated with cold response, although the underlying mechanisms are under investigation [66,67,68].

The polygenic genetic architecture of frost resistance traits encourages the use of genomic selection to account for the effect of many genes in linkage with SNPs. The presence of many relevant genes with a minor effect was also supported by the minimal or nil decrease in predictive ability observed for the model constructed from landrace and cultivar data when passing from intra-population prediction to cross-population prediction, in the presence of marked inconsistency between the two reference populations for significant SNPs in the GWAS. Our results confirmed the interest of genomic prediction models constructed for a genetically broad reference population, such as the world collection of landrace and cultivar materials, not only to identify promising genetic resources within large germplasm collections but also for a preliminary genomic selection of breeding lines. Within cool-season grain legumes, the transferability to a breeding line population of genomic prediction models constructed from data of a world germplasm collection have already emerged for seed weight and oil content in white lupin [42] and seed protein content and seed weight in pea [36], albeit at the cost of a predictive ability drop in the range of 35–50% relative to intra-population prediction of the breeding lines. The current predictive ability drop of 41–43% agrees well with those findings. Hence, even in favorable cases, the model transferability is likely to be envisaged only when budget constraints impede the development of a specific prediction model for the targeted breeding line population. Of course, a lack of model transferability may emerge, as in the case of seed protein content of white lupin [42] and a highly complex trait such as pea grain yield [36]. The modest cross-population predictive ability exhibited by the model constructed using reference population 2 to predict reference population 1 confirmed earlier results [42] showing that a training reference population with relatively low genetic variation (such as that of the breeding lines) restricts the inference space for the prediction of breeding values in a genetically broad, independent reference population.

The intra-population scenario for the breeding lines showed high predictive abilities (in the range of 0.67–0.68). This result aligns with previous results obtained for frost biomass injury of cereal breeding populations, which showed a prediction accuracy of 0.59 for wheat [69] and a predictive ability of 0.87 for rye [70]. In a marker-assisted selection prospect, a marker score was able to explain about 30% of the variation for faba bean resistance to winter and late-winter frosts [71]. The currently high predictive ability values encourage the prediction of frost resistance aimed to restrict the number of breeding lines subjected to platform-based and/or field-based evaluation. The predictive ability value of 0.41 observed for intra-population prediction of plant mortality in the international collection of landraces and cultivars, while being expectedly lower than the value for breeding lines as a consequence of the wider genetic variation and faster LD of this material, is nevertheless useful for the identification of genetic resources with putative frost resistance to be verified by subsequent evaluation. The use of genomic prediction models for white lupin germplasm collections is justified by their large number of accessions, which exceeds 6300 just considering the main collections [72] and prevents a thorough evaluation due to limited budgets.

In conclusion, this study can support the selection of winter-hardy white lupin cultivars targeted to autumn-sown environments by (a) highlighting the extent of genetic variation for frost resistance, (b) elucidating the impact of frost resistance and a late phenology on field-based winter plant survival, (c) identifying frost-resistant genetic resources, (d) showing the definite polygenic architecture of frost resistance, (e) revealing genomic regions hosting putative candidate genes, and (f) highlighting opportunities for genomic prediction models aimed at the selection of breeding lines or the identification of promising genetic resources. Our study indicated that the genomic selection of inbred lines can be valuable even via the cross-population prediction of models constructed from a genetically broad reference population such as a world germplasm collection, albeit less effective than intra-population prediction. The overall contribution of frost resistance on field-based winter survival was modest in the presence of large variation for the onset of flowering but increased substantially for a material with a similar phenology, emphasizing the need to combine selection for both frost resistance and a sufficient vernalization requirement. The highly positive impact of the late onset of flowering on winter plant survival, and the highly negative impact of the same trait on tolerance to terminal drought in earlier work [17,18], highlight the strategic importance to identify a convenient, intermediate vernalization requirement on the one hand and to exploit the genetic variation for resistance to frost and to terminal drought on the other, when breeding for autumn sowing in cold- and drought-prone regions. Genomic selection models for drought resistance, which have already been defined for breeding line [18] and landrace material [32], could be used in combination with the current models to select simultaneously for frost and drought resistance. Evaluation under controlled conditions is promising for both germplasm selection and the refinement of genomic prediction models.

## 4. Materials and Methods

### 4.1. Plant Material

Our study included two white lupin populations, one comprising landrace and cultivar genotypes (reference population 1), and the other including sweet-seed inbred lines from a breeding program (reference population 2). Reference population 1 consisted of 127 genotypes belonging to 107 landraces, along with 17 commercial cultivars, for a total number of 144 evaluated genotypes whose origins are given in Table S1. The landraces were selected from a world collection described in [15] and belonged to 11 regional germplasm pools reflecting the main historical white lupin cropping regions. Each accession was represented by at least one randomly chosen individual genotype, but two genotypes per accession were included in some cases. Genotypes selected from the same landrace were genetically distinct, as confirmed by the observed molecular marker diversity and consistent with the expected intra-landrace variation. The 17 commercial cultivars were classified into three phenological classes, namely (a) spring types, featuring nil or minimal vernalization requirement and adapted to early-spring sowing in cold-prone areas of northern or continental Europe; (b) Mediterranean types, with an intermediate vernalization requirement and adapted to autumn sowing in Mediterranean regions; and (c) winter types, with a high vernalization requirement and adapted to autumn sowing in regions with a subcontinental or suboceanic climate [29,45]. All landraces, along with eight cultivars, had previously been evaluated for field-based winter plant survival and onset of flowering in Lodi (northern Italy), an environment characterized by a subcontinental climate, where the plants were exposed to an absolute minimum temperature of −9 °C [15].

Reference population 2 consisted of sweet-seed breeding lines originating from 16 crosses produced by a 4 × 4 factorial mating design. Each of four elite, sweet-seed cultivars or breeding lines, namely, the French cultivar Lucky, the French breeding line MB-38, the Italian variety Arsenio, and the Moroccan breeding line L27PS3, was crossed with each of four elite, bitter-seed landrace accessions, namely, the Italian landraces LAP123 and La246, the landrace La646 from the Canary Islands, and the Greek landrace Gr56. The selection of parent germplasm, which was based on desirable agronomical traits, and the development of inbred lines until the F_6_ seed under insect-proof cages (to prevent cross-pollinations), were described earlier [18]. The landrace Gr56 showed high winter hardiness in previous work [15]. The lines underwent selection for low alkaloid content in F_3_ and F_4_ generations [42]. Between four and eleven lines per cross contributed to the current population of 144 test lines. The number of lines issued from each of the eight parents was moderately balanced, ranging from 30 for MB-38 to 39 for L27PS3. The full list of breeding lines is provided in Table S2 along with the indication of the parent germplasm. All landraces used as parental germplasm were also tested as single genotypes in reference population 1.

### 4.2. Phenotyping

The two populations were evaluated under two separate experiments performed in a high-throughput phenotyping platform according to a protocol for frost resistance assessments that was defined in a prior methodological study [19] (in which frost resistance was termed as intrinsic frost tolersnce). In brief, the platform consisted of a freezing growth chamber equipped with LED lamps. With a surface area of 13.63 m^2^, it could accommodate up to 2160 plants placed in polystyrene plug trays with cells measuring 5 cm × 5 cm × 15 cm in depth. The trays were filled with a commercial growing substrate composed of pH-adjusted peat (pH = 6.0) and NPK mineral fertilizer (substrate SER CA-V7, Vigorplant, Piacenza, Italy), and were arranged side by side on four large trolleys. Each experiment was set up as an alpha lattice with 12 incomplete blocks of 12 experimental units each, and 4 replications. Each growing cycle comprised one replication. Each experiment unit included 15 plants of the relevant genotype.

The seeds were pre-germinated on filter paper in Petri dishes for approximately 48 h at 19 °C before being transplanted into the plug trays at a depth of 2.5 cm. The protocol included (i) 10 days of growth at 22.5 °C with 12 h of daylight, (ii) 15 days of cold acclimation (hardening) at 4 °C with 10 h of daylight, (iii) 12 h of cooling at −3 °C in the dark, (iv) 4 h of freezing treatment at −11 °C, (v) 2 days of recovery at 4 °C with 10 h of daylight, and (vi) 19 days of regrowth at 15/20 °C (night/day) with 12 h of daylight (Figure S5). The freezing temperature of −11 °C was identified as the one with the highest screening value for the species [19]. The temperature decreased toward the freezing point and then increased at a rate of 1 °C/h. Plants were irrigated every two days during growth, recovery, and regrowth, while suspending the irrigationduring hardening and the freezing treatment.

**Figure 5:**
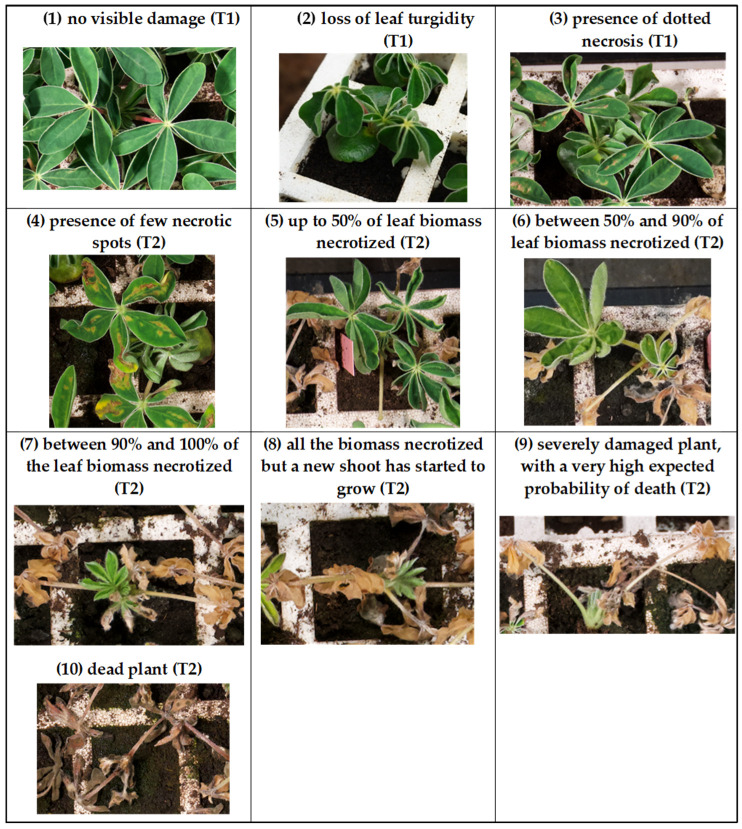
Ten-level visual score of aerial biomass frost injury based on observations on each individual plant carried out after six days (T1) or three weeks (T2) after frost exposure.

The frost resistance of the genotypes was evaluated by two traits: (a) the plant mortality proportion; (b) a visual score of aerial biomass injury. The latter trait was assessed on individual plants, and comprised the 10 levels of increasing damage described in Figure 5. This assessment was based on two observation timepoints: one six days after frost, when mild injuries are more evident; the other at the end of regrowth for severe damage and mortality observations, which can be accurately assessed after a minimum period of three weeks [73]. Scores on individual plants were averaged across the experimental unit. Plants scoring 9 and 10 were classified as dead for mortality assessment.

### 4.3. Phenotypic Data Analysis

Plant mortality and visually scored biomass injury were analyzed separately for each reference population according to the following linear mixed model:

$$Y_{irb}\;=\;\mu \;+\;G_{i}\;+\;{Ρ}_{r}+\;{Β}_{rb}+\;E_{irb};\;E_{irb}\;\sim \;N(0,\;\sigma_{e}^{2})$$

where the response variable $Y_{irb}\;$ is a function of the trait mean *μ*, genotype ($G_{i}$) and replicate (${Ρ}_{r}$) effects treated as fixed factors, a block within replicate random effect ${(Β}_{rb})$, and with $E_{irb}\;$ representing the model residual. The significance of each source of variation was assessed by analysis of variance (ANOVA). Lattice-adjusted genotype mean values of materials belonging to reference population 1 underwent a second ANOVA including the germplasm pool as a fixed factor and the genotype within the pool as a random factor (hence, acting as an error term for germplasm pools). We assessed the consistency of frost resistance response between landrace parent material (belonging to reference population 1) and their progenies (averaged across relevant breeding lines of reference population 2). Genotype mean values of landraces and cultivars that were previously evaluated for field-based winter plant mortality [15] underwent a linear regression analysis of field mortality as a function of mortality under controlled conditions. Values in the latter condition of genotypes belonging to the same landrace were averaged before the analysis. Landraces and cultivars were graphically grouped into three classes of onset of flowering, namely, early flowering (18 entries), intermediate flowering (76 entries), and late flowering (21 entries), based on their flowering date (measured in days from January 1 to the point when 50% of the plants had at least one flower open). The accessions flowering earlier than the mean minus 0.5 × standard deviation were classified as early flowering; those later than the mean plus 0.5 × standard deviation were classified as late flowering; and the remaining ones were classified as intermediate.

The model above, but holding genotype ($G_{i}$) as a random factor, was used for estimating genotype ($\sigma_{g}^{2}$) and experiment error ($\sigma_{e}^{2}$) variance components by the restricted maximum likelihood (REML) method. This analysis aimed to compute the genetic coefficient of variation $\left({CV}_{g}\right)$ and broad-sense heritability on an entry mean basis of ($H^{2}$) values as follows:

$${CV}_{g}=\;(\sigma_{g}/\;\mu )\;\times \;100$$

$$H^{2}=\frac{\sigma_{g}^{2}}{\left(\sigma_{g}^{2}+\frac{\sigma_{e}^{2}}{n}\right)}$$

where $\sigma_{g}$ is the square root of the genotype variance component, and *n* is the number of experiment replications. We used *H*^2^ values to compute best linear unbiased prediction (BLUP) values of genotypes according to [74]. These values were used to calculate Pearson correlation coefficients (*r*) between traits, and acted as phenotypic data for trait-marker analyses and genomic predictions.

All statistical analyses were performed with the statistical software R (version 4.4.1; R Core Team). Models were fitted by the *lm()* and *lmer()* functions from the R-packages “stats” -and “lme4”, respectively.

### 4.4. DNA Isolation, GBS Library Construction, and Sequencing

Genomic DNA was extracted from young leaves of landrace and cultivar genotypes (reference population 1) and from F_5_ plants of each breeding line (reference population 2) using the DNeasy Plant Mini Kit (Qiagen, Milan, Italy). A Quant-iTTM PicoGreenTM dsDNA Assay Kit (P7589, Life Technologies, Trieste, Italy) was used to quantify the nucleic acid, and 1% agarose gel electrophoresis was used to verify its quality. A trial digestion was performed on 10% of the DNA samples using the Optizyme EcoRI restriction enzyme (25,000 U, Fisher BioReagents, Rodano, Italy) to compare bands of cut and uncut DNA. The reaction was performed at 37 °C for an hour and the enzyme was deactivated at 65 °C for 20 min. DNA samples were sent to The Elshire Group Ltd. laboratory (Palmerston North, New Zealand) for outsourced library preparation and sequencing. GBS data were generated according to [30], with the following modifications: 100 ng of genomic DNA, and 3.6 ng of total adapters were used, and the genomic DNA was restricted with ApeKI enzyme (NEB New England Biolabs, R0643L, Ipswich, MA, USA); the library was then amplified using Kapa Taq polymerase Alpha (KAPA Library Amplification Readymix, Kapa Biosystems KK2611, Cape Town, South Africa) by 14 PCR cycles. Sequencing was performed on a single Illumina HiSeq X lane, at 2 × 150 bp paired end. The adoption of ApeKI as the restriction enzyme, according to [30], was supported by the fact that about 60% of the white lupin genome includes repetitive DNA sequences [37], which this enzyme tends to skip.

### 4.5. Genotype SNP Calling Procedures, Data Filtering and Imputation

SNP calling was based on Legpipe2 pipeline default settings for diploid species [75]. For alignment, we used the *Lupinus albus* genome version 1.0 [37], which was downloaded from https://www.whitelupin.fr/ (accessed on 4 August 2025). The entire dataset, comprising landrace and cultivar genotypes (reference population 1) and breeding lines (reference population 2) was filtered for monomorphic markers, with a missing rate per marker < 1%, a missing rate per individual < 10%, and SNP heterozygosity < 30%. Due to relevant differences in allele frequency among reference populations 1 and 2, the two datasets were filtered separately for minor allele frequency > 5%. This process retained 40,914 SNPs for landrace and cultivar genotypes (reference population 1) and 32,951 SNPs for breeding lines (reference population 2). We used random forest imputation to estimate missing data [76], using the R package MissForest [77], with the configuration ntree = 100, maxiter = 10, and encoding genotypes as categorical data (factors).

### 4.6. Analysis of Linkage Disequilibrium Decay, Population Structure, and Genome-Wide Association Study

The analyses of LD decay and population structure, and the GWAS, were performed separately for the two reference populations. LD was estimated per chromosome as *r*^2^ values for pairwise combinations of SNPs within a 100 kb window using the *LD.decay()* function from the R package “sommer” [78]. The *r*^2^ values were plotted against physical distance and fitted by a polynomial curve, as described in [79]. The critical value of *r*^2^, representing the most meaningful LD decay threshold, was derived from the distribution of inter-chromosomal LD (distribution of *r*^2^ estimates derived from SNPs located on different chromosomes), as described in [80]. Inter-chromosomal *r*^2^ estimates were square-root transformed to approximate a normally distributed random variable. The parametric 95th percentile of that distribution was assumed as a population-specific critical value of *r*^2^ above which LD was likely attributable to physical linkage. For each chromosome, the LD extent was estimated as the physical distance at which the fitted polynomial curve of decay crossed the population-specific critical *r*^2^ threshold.

The population structure was investigated by DPCA [81] performed on genotype data previously pruned for the excess of linkage disequilibrium, to avoid the strong influence of SNP clusters when estimating genetic relatedness [82]. Pruning was performed by the *snpgdsLDpruning()* function from the R package “SNPRelate” with a maximum *r*^2^ threshold of 0.45. The k-means clustering algorithm was run iteratively for increasing values of K from 1 to 20 and repeated 300 times to identify the optimal numbers of genotype groups (K) based on the minimization of the Bayesian information criterion. The analysis was performed on the output of an ordinary principal component analysis (PCA) to benefit from dimensionality reduction, but all components were retained to avoid information loss. The final DPCA was carried out according to the optimal K value, which resulted equal to 2 for reference population 1 and 19 for reference population 2. The number of principal component (PC) axes retained for DPCA was determined by visual inspection of the plots of PC cumulative variance (100 PCs for reference population 1, and 80 PCs for reference population 2). The number of discriminant functions to be employed as covariates in the GWAS was determined by the a-score optimization criterion (which represents the propensity of DPCA toward overfitting). Based on this criterion, one discriminant function was retained for reference population 1, and 13 for reference population 2. The entire procedure was implemented using the functions *find.clusters()*, *dapc()*, and *oprim.a.score()* from R package “adegenet” [83].

A GWAS was conducted for plant mortality and the visual injury score within each reference population according to the Blink model [84] using the R package “GAPIT” [85]. We accounted for the population structure by including the discriminant functions obtained by DPCA as model covariates. A visual examination of quantile–quantile (QQ) plots (Figure S6) confirmed an appropriate compensation of population structure in the GWAS models. Significant SNPs were selected according to the FDR threshold at 5%. Based on the previously computed LD extent within each chromosome, candidate genes in association with the significant SNPs and corresponding putative functions were identified on the white lupin genome browser (www.whitelupin.fr) by scanning a genomic region corresponding to the chromosome-specific LD extent in both directions from each significant SNP.

### 4.7. Genomic Selection

Genomic prediction models were assessed for plant mortality and the visual injury score using the R package “GROAN” [86]. We envisaged an intra-population prediction scenario for each population by comparing three statistical models (described below), which included all available SNPs (40,914 for reference population 1, and 32,951 for reference population 2). The predictive ability (i.e., the correlation between observed and predicted phenotypes) was estimated through a standard 10-fold cross validation repeated 10 times to ensure numerical stability (reporting the average results). The transferability of the predictive models in a cross-population scenario was investigated by fitting each of the three statistical models on reference population 1 and validating them on reference population 2, and vice versa. Only markers common to both populations were included in this analysis, namely a total of 30,412 SNPs.

The statistical models involved in this study were ridge regression BLUP (rrBLUP), Bayesian Lasso (BL), and BayesB. The rrBLUP model [87] assumes a linear mixed additive model, where each marker is assigned an effect as a solution of the following equation:

y=1µ+Wq+E

where y is the vector of observed phenotypes, *µ* is the mean of y, *W* is the genotype matrix (e.g., {0,1,2} for biallelic SNPs), *q* ∼ N (0, ${I\sigma }_{q}^{2}$) is the vector of marker effects, and ε ∼ N (0, ${I\sigma }_{e}^{2}$) is the vector of residuals. This model, which is solved in a restricted maximum likelihood (REML) context, assumes that the effects of all loci have a common variance, making it suitable for traits influenced by a large number of minor genes.

BL and BayesB models fit the same general model as rrBLUP but within a Bayesian context, where different prior densities are assigned to marker effects, allowing them to have different variances [88,89]. BL assumes independent Laplace double-exponential priors, which impose a strong shrinkage for regression coefficients of marker effects with small values [90]. Bayes B assumes that marker effects follow a *t*-distribution and that most loci have no effect on the trait and are, therefore, excluded from the prediction model [88]. Bayesian models use Markov chain Monte Carlo to estimate the model parameters (thereby introducing Monte Carlo sampling error as a source of variation). To ensure numerical stability of the results for models fit in one population and validation in the other, the number of iterations of the algorithm was set to 50,000.

## Figures and Tables

**Table 1:** Number of evaluated genotypes and mean and range values of proportion of plant mortality and visual score of biomass injury assessed at −11 °C freezing temperature in a phenotyping platform, for white lupin pools of landrace and cultivar genotypes.

Pool	Material	No. of Genotypes	Mortality		Visual Score	
			Mean ^a^	Range	Mean ^a^	Range
Winter-type	Cultivar	4	0.21 d	0.09–0.37	5.6 d	4.8–6.3
Mediterranean-type	Cultivar	5	0.33 cd	0.13–0.58	6.2 cd	4.4–7.5
Greece	Landrace	12	0.44 bc	0.08–0.70	7.0 bc	5.3–8.6
Spain	Landrace	11	0.48 bc	0.17–0.70	7.1 bc	5.7–8.3
Madeira and Canaries	Landrace	10	0.51 bc	0.27–0.73	7.3 b	6.2–8.5
Azores	Landrace	11	0.51 bc	0.18–0.80	7.2 b	5.1–8.7
West Asia	Landrace	12	0.52 ab	0.26–0.76	7.3 b	5.9–8.8
Turkey	Landrace	12	0.53 ab	0.16–0.73	7.5 b	6.1–8.9
Italy	Landrace	14	0.54 ab	0.26–0.75	7.5 ab	5.7–8.7
Portugal	Landrace	10	0.55 ab	0.25–0.76	7.7 ab	6.6–8.7
Egypt	Landrace	14	0.56 ab	0.16–0.82	7.7 ab	5.2–9.0
East Africa	Landrace	10	0.57 ab	0.21–0.95	7.6 ab	5.5–9.4
Spring-type	Cultivar	8	0.63 ab	0.12–0.90	7.9 ab	4.9–9.5
Maghreb	Landrace	11	0.71 a	0.45–0.96	8.4 a	7.4–9.7
LSD (*p* < 0.05)			0.16		0.9	

^a^ Means followed by different letters differ at *p* < 0.05 according to Duncan’s test.

**Table 2:** Mean values of proportion of plant mortality and visual score of biomass injury assessed at −11 °C freezing temperature in a phenotyping platform for four white lupin parental landraces and the mean values of their progeny lines.

Landrace	Mortality		Visual Score	
	Parent Value	Progeny Value	Parent Value	Progeny Value
Gr56	0.24	0.49	6.0	7.6
La646	0.39	0.57	6.6	8.0
La246	0.36	0.66	6.4	8.5
LAP123	0.72	0.77	8.6	8.9
LSD (*p* < 0.05)	0.34	0.06	1.5	0.6

**Table 3:** List of candidate genes associated with significant SNPs detected by a GWAS. The analysis was conducted on white lupin landrace and cultivar genotypes (reference population 1) and breeding lines (reference population 2), for the proportion of plant mortality and a visual score of biomass injury assessed at −11 °C freezing temperature in a phenotyping platform. Candidate genes were identified using the white lupin genome browser (www.whitelupin.fr; accessed on 4 August 2025). Putative encoded proteins and their putative roles in frost resistance are reported.

SNP	Population	Trait	Candidate Gene	Putative Protein	Putative Role
Chr02_14306413	2	Mortality, visual score	Chr02g0156401	Metallo-dependent phosphatase	Cold signal regulation
Chr02_14306413	2	Mortality, visual score	Chr02g0156391	Multi antimicrobial extrusion protein	
Chr02_14306413	2	Mortality, visual score	Chr02g0156411	UDP-N-acetylglucosamine--dolichyl-phosphate N-acetylglucosaminephosphotransferase	
Chr04_7632627	2	Mortality	Chr04g0256531	Hydrolase	Stabilization of cell wall
Chr04_7632627	2	Mortality	Chr04g0256541	Potassium transporter	Cryoprotection and osmoprotection
Chr05_4820341	1	Mortality	Chr05g0219341	CBS domain-containing protein (CDCPs)	
Chr05_4820341	1	Mortality	Chr05g0219331	Polyadenylate binding protein, human types 1, 2, 3, 4	
Chr06_1878682	1	Mortality	Chr06g0163651	Oxidoreductase	Enhancement of ROS scavenging
Chr06_1878682	1	Mortality	Chr06g0163661	mRNA splicing factor SYF2	
Chr08_3511620	1	Mortality	Chr08g0234251	Cellulose synthase (UDP-forming) chromatin regulator PHD family	Gene expression regulation
Chr08_3511620	1	Mortality	Chr08g0234241	QWRF family protein	
Chr13_15386976	1	Mortality	Chr13g0303311	Ribosomal protein S4/S9	Ribosomal biogenesis
Chr13_15386976	1	Mortality	Chr13g0303301	Transcription factor bHLH family	Cryoprotection and osmoprotection
Chr14_10127694	1	Mortality	Chr14g0368501	Leucine-rich repeat domain, L domain-containing protein	Primary cold sensor
Chr14_10127694	1	Mortality	Chr14g0368511	Plus-end-directed kinesin ATPase	
Chr16_5032297	1	Visual score	Chr16g0384801	Kinase RLK-Pelle-URK-1 family	Primary cold sensor
Chr19_17886074	2	Mortality	Chr19g0139891	Vacuolar protein sorting-associated protein	
Chr19_17886074	2	Mortality	Chr19g0139901	Methionine--tRNA ligase	
Chr19_17886074	2	Mortality	Chr19g0139881	Phosphatase 4 core regulatory subunit R2	
Chr21_1050253	2	Mortality	Chr21g0306351	Serine/threonine phosphatase, protein kinase CMGC-GSKL family	Cold signal regulation
Chr23_1146188	1	Mortality, visual score	-	-	

**Table 4:** Predictive ability (as a correlation between observed and predicted phenotypes) of the best-performing of three statistical models (rrBLUP, Bayesian Lasso, BayesB) for the proportion of plant mortality and the visual score of biomass injury assessed at −11 °C freezing temperature in a phenotyping platform in two white lupin reference populations (population 1: landrace and cultivar genotypes; population 2: breeding lines). Predictions for intra-population and cross-population scenarios.

Trait	Training Set	Validation Set	Model	Predictive Abilities
Mortality	Population 1	Population 1	rrBLUP	0.414
Mortality	Population 2	Population 2	Bayesian Lasso	0.672
Mortality	Population 1	Population 2	Bayesian Lasso	0.393
Mortality	Population 2	Population 1	Bayesian Lasso	0.255
Visual score	Population 1	Population 1	rrBLUP	0.376
Visual score	Population 2	Population 2	Bayesian Lasso	0.678
Visual score	Population 1	Population 2	Bayesian Lasso	0.386
Visual score	Population 2	Population 1	Bayesian Lasso	0.232

## Data Availability

The genotypic data used for this study are available in the Figshare repository at https://doi.org/10.6084/m9.figshare.30146242.v1, whereas phenotyping data are reported as Supplementary Materials.

## Supplementary Materials

The following supporting information can be downloaded at https://www.mdpi.com/article/10.3390/ijms262010224/s1.

## Abbreviations

The following abbreviations are used in this manuscript:

GBS	Genotyping-by-sequencing
SNP	Single-Nucleotide Polymorphism
FDR	False discovery rate
LD	Linkage disequilibrium
ANOVA	Analysis of variance
BLUE	Best linear unbiased estimate
BLUP	Best linear unbiased prediction
rrBLUP	Ridge regression best linear unbiased prediction
BL	Bayesian Lasso
