# Supplementary material for: Genetic Variation and Genome-Enabled Prediction of White Lupin Frost Resistance in Different Reference Populations
Source: Int J Mol Sci. 2025 Oct 21;26(20):10224. doi: 10.3390/ijms262010224 (PMC12563025; doi:10.3390/ijms262010224)
Supplement: Supplementary file 1 [file ijms-26-10224-s001.zip › ijms-3912098-supplementary.pdf]

Table S1. List of 144 white lupin landrace and cultivar genotypes (reference population 1). For each genotype, the following information is reported: name, original accession name, type of material (landrace or cultivar), corresponding germplasm pool, origin, mean values of plant mortality proportion and visual score of biomass injury assessed at −11°C freezing temperature in a phenotyping platform, and availability of field data (mortality and onset of flowering) associated with the accession.

Genotype	Accession	Material	Germplasm pool	Origin	Mortality	Visual Score	Field data
LAP0001b	Ac003	Landrace	Azores	Azores	0.18	5.1	Yes
LAP002acd	Ac045	Landrace	Azores	Azores	0.44	6.8	Yes
LAP0003c	Ac050	Landrace	Azores	Azores	0.67	8.0	Yes
LAP0005a	Ac079	Landrace	Azores	Azores	0.49	7.2	Yes
LAP0005d	Ac079	Landrace	Azores	Azores	0.54	7.1	Yes
LAP006bc	Ac085	Landrace	Azores	Azores	0.46	7.2	Yes
LAP0007c	Ac139	Landrace	Azores	Azores	0.33	6.9	Yes
LAP0007a	Ac139	Landrace	Azores	Azores	0.80	8.7	Yes
LAP0008abc	Ac140	Landrace	Azores	Azores	0.64	8.2	Yes
LAP0009ab	Ac155	Landrace	Azores	Azores	0.57	7.4	Yes
LAP0010bc	Ac156	Landrace	Azores	Azores	0.48	6.8	Yes
LAP0078ac	La020	Landrace	East Africa	Ethiopia	0.65	8.1	Yes
LAP0080a	La399	Landrace	East Africa	Ethiopia	0.67	8.2	Yes
LAP0081bc	La420	Landrace	East Africa	Sudan	0.48	6.9	Yes
LAP0082d	La422	Landrace	East Africa	Sudan	0.39	6.7	Yes
LAP0079d	La559	Landrace	East Africa	Ethiopia	0.95	9.4	Yes
LAP0083a	La629	Landrace	East Africa	Sudan	0.83	9.0	Yes
LAP0075b	La654	Landrace	East Africa	Kenya	0.21	5.5	Yes
LAP0075a	La654	Landrace	East Africa	Kenya	0.27	6.3	Yes
LAP0076b	La655	Landrace	East Africa	Kenya	0.80	9.0	Yes
LAP0077a	La656	Landrace	East Africa	Kenya	0.46	6.7	Yes
LAP0085d	Egypte003	Landrace	Egypt	Egypt	0.82	9.0	Yes
LAP0086d	Egypte011	Landrace	Egypt	Egypt	0.48	6.8	Yes
LAP0088a	Egypte016	Landrace	Egypt	Egypt	0.26	5.7	Yes
LAP0089b	Egypte022	Landrace	Egypt	Egypt	0.62	8.5	Yes
LAP0087d	Egypte026	Landrace	Egypt	Egypt	0.64	8.1	Yes
LAP0090bd	Egypte038	Landrace	Egypt	Egypt	0.68	8.2	Yes
LAP0091b	Egypte055	Landrace	Egypt	Egypt	0.57	7.7	Yes
LAP0091a	Egypte055	Landrace	Egypt	Egypt	0.59	8.2	Yes
LAP0092a	Egypte064	Landrace	Egypt	Egypt	0.16	5.2	Yes
LAP0093c	Egypte076	Landrace	Egypt	Egypt	0.75	8.6	Yes
LAP0094b	Egypte093	Landrace	Egypt	Egypt	0.33	6.3	Yes
LAP0094d	Egypte093	Landrace	Egypt	Egypt	0.68	8.4	Yes
LAP0095c	La356	Landrace	Egypt	Egypt	0.49	7.5	Yes
LAP0096a	La364	Landrace	Egypt	Egypt	0.78	9.0	Yes
LAP0011cd	Gr003	Landrace	Greece	Greece	0.48	7.4	Yes
LAP0012c	Gr005	Landrace	Greece	Greece	0.50	7.3	Yes
LAP0013c	Gr017	Landrace	Greece	Greece	0.47	6.8	Yes
LAP0014ad	Gr021	Landrace	Greece	Greece	0.58	7.9	Yes
LAP0015b	Gr025	Landrace	Greece	Greece	0.34	6.6	Yes
LAP0016a	Gr030	Landrace	Greece	Greece	0.57	7.7	Yes

LAP0017b	Gr033	Landrace	Greece	Greece	0.56	7.8	Yes
LAP0017a	Gr033	Landrace	Greece	Greece	0.70	8.6	Yes
LAP0018a	Gr049	Landrace	Greece	Greece	0.31	6.7	Yes
LAP0019d	Gr056	Landrace	Greece	Greece	0.08	5.3	Yes
LAP0019c	Gr056	Landrace	Greece	Greece	0.39	6.6	Yes
LAP0020a	Gr057	Landrace	Greece	Greece	0.23	5.4	Yes
LAP0097a	Ita001	Landrace	Italy	Italy	0.54	7.7	Yes
LAP0098ac	Ita002	Landrace	Italy	Italy	0.54	7.5	Yes
LAP0103c	Ita020	Landrace	Italy	Italy	0.26	5.7	Yes
LAP0099c	Ita037	Landrace	Italy	Italy	0.70	8.3	Yes
LAP0100a	Ita042	Landrace	Italy	Italy	0.71	8.2	Yes
LAP0104b	Ita049	Landrace	Italy	Italy	0.34	6.8	Yes
LAP0104d	Ita049	Landrace	Italy	Italy	0.55	7.7	Yes
LAP0101c	Ita051	Landrace	Italy	Italy	0.55	7.3	Yes
LAP0105abd	Ita057	Landrace	Italy	Italy	0.34	6.3	Yes
LAP0106c	Ita058	Landrace	Italy	Italy	0.56	7.2	Yes
LAP0107a	La246	Landrace	Italy	Italy	0.36	6.4	Yes
LAP0123b	LAP123	Landrace	Italy	Italy	0.69	8.4	Yes
LAP0123c	LAP123	Landrace	Italy	Italy	0.75	8.7	Yes
LAP0124c	LAP124	Landrace	Italy	Italy	0.67	8.6	Yes
LAP0057b	La197	Landrace	Madeira & Canaries	Madeira	0.63	8.0	Yes
LAP0058d	La198	Landrace	Madeira & Canaries	Madeira	0.73	8.5	Yes
LAP0059b	La641	Landrace	Madeira & Canaries	Canaries	0.27	6.2	Yes
LAP0059c	La641	Landrace	Madeira & Canaries	Canaries	0.48	6.9	Yes
LAP0060abc	La642	Landrace	Madeira & Canaries	Canaries	0.61	7.9	Yes
LAP0061d	La646	Landrace	Madeira & Canaries	Canaries	0.36	6.6	Yes
LAP0061c	La646	Landrace	Madeira & Canaries	Canaries	0.42	6.7	Yes
LAP0062c	La648	Landrace	Madeira & Canaries	Canaries	0.50	7.4	Yes
LAP0063bd	La652	Landrace	Madeira & Canaries	Canaries	0.57	7.4	Yes
LAP0064b	La653	Landrace	Madeira & Canaries	Canaries	0.51	7.2	Yes
LAP0054a	La060	Landrace	Maghreb	Morocco	0.89	9.3	Yes
LAP0049ad	La127	Landrace	Maghreb	Algeria	0.66	8.2	Yes
LAP0055ac	La150	Landrace	Maghreb	Morocco	0.89	9.2	Yes
LAP0050bcd	La568	Landrace	Maghreb	Algeria	0.62	7.9	Yes
LAP0051d	La572	Landrace	Maghreb	Algeria	0.58	7.4	Yes
LAP0051b	La572	Landrace	Maghreb	Algeria	0.64	8.3	Yes
LAP0056c	La680	Landrace	Maghreb	Morocco	0.63	7.6	Yes
LAP0052d	La686	Landrace	Maghreb	Algeria	0.96	9.7	Yes
LAP0053b	La688	Landrace	Maghreb	Algeria	0.71	8.6	Yes
LAP0109d	Maroc74	Landrace	Maghreb	Morocco	0.45	7.6	Yes
LAP0110ad	Maroc78	Landrace	Maghreb	Morocco	0.77	8.8	Yes
LAP0041abc	E080	Landrace	Portugal	Portugal	0.53	7.4	Yes
LAP0042c	E091	Landrace	Portugal	Portugal	0.67	8.3	Yes
LAP0042b	E091	Landrace	Portugal	Portugal	0.76	8.7	Yes
LAP0043d	E099	Landrace	Portugal	Portugal	0.68	8.5	Yes
LAP0044b	E104	Landrace	Portugal	Portugal	0.68	7.9	Yes
LAP0045a	E107	Landrace	Portugal	Portugal	0.25	7.2	Yes

LAP0046a	E112	Landrace	Portugal	Portugal	0.53	7.4	Yes
LAP0047a	E126	Landrace	Portugal	Portugal	0.40	6.6	Yes
LAP0047c	E126	Landrace	Portugal	Portugal	0.46	7.2	Yes
LAP0048bc	E132	Landrace	Portugal	Portugal	0.53	7.4	Yes
LAP0031acd	E002	Landrace	Spain	Spain	0.44	6.7	Yes
LAP0032bd	E003	Landrace	Spain	Spain	0.48	7.2	Yes
LAP0033d	E016	Landrace	Spain	Spain	0.27	5.9	Yes
LAP0034c	E036	Landrace	Spain	Spain	0.45	6.7	Yes
LAP0034a	E036	Landrace	Spain	Spain	0.61	7.9	Yes
LAP0035ab	E059	Landrace	Spain	Spain	0.67	8.2	Yes
LAP0036c	E064	Landrace	Spain	Spain	0.17	5.7	Yes
LAP0036a	E064	Landrace	Spain	Spain	0.70	8.3	Yes
LAP0037d	E068	Landrace	Spain	Spain	0.63	7.7	Yes
LAP0039c	E075	Landrace	Spain	Spain	0.50	7.3	Yes
LAP0040c	E191	Landrace	Spain	Spain	0.39	6.6	Yes
LAP0027a	La110	Landrace	Turkey	Turkey	0.71	8.4	Yes
LAP0028d	La120	Landrace	Turkey	Turkey	0.73	8.9	Yes
LAP0029a	La259	Landrace	Turkey	Turkey	0.56	7.7	Yes
LAP0030c	La431	Landrace	Turkey	Turkey	0.32	6.5	Yes
LAP0021ad	Tr001	Landrace	Turkey	Turkey	0.54	7.2	Yes
LAP0022e	Tr007	Landrace	Turkey	Turkey	0.43	6.7	Yes
LAP0022b	Tr007	Landrace	Turkey	Turkey	0.54	7.0	Yes
LAP0023b	Tr012	Landrace	Turkey	Turkey	0.16	6.1	Yes
LAP0024b	Tr016	Landrace	Turkey	Turkey	0.55	8.0	Yes
LAP0025a	Tr017	Landrace	Turkey	Turkey	0.56	7.6	Yes
LAP0025b	Tr017	Landrace	Turkey	Turkey	0.72	8.8	Yes
LAP0026d	Tr021	Landrace	Turkey	Turkey	0.53	7.4	Yes
LAP0071b	La406	Landrace	West Asia	Israel	0.50	7.4	Yes
LAP0072a	La409	Landrace	West Asia	Israel	0.72	8.3	Yes
LAP0073b	La415	Landrace	West Asia	Jordan	0.40	6.5	Yes
LAP0068d	La416	Landrace	West Asia	Lebanon	0.39	6.4	Yes
LAP0068c	La416	Landrace	West Asia	Lebanon	0.45	6.8	Yes
LAP0069bc	La418	Landrace	West Asia	Lebanon	0.59	8.0	Yes
LAP0065a	La425	Landrace	West Asia	Syria	0.76	8.8	Yes
LAP0066bcd	La427	Landrace	West Asia	Syria	0.53	7.1	Yes
LAP0074c	La432	Landrace	West Asia	Jordan	0.71	8.1	Yes
LAP0067d	La547	Landrace	West Asia	Syria	0.26	5.9	Yes
LAP0067b	La547	Landrace	West Asia	Syria	0.64	8.0	Yes
LAP0070abcd	La673	Landrace	West Asia	Lebanon	0.29	6.2	Yes
LAP0113ab	Amiga	Cultivar	Spring-type	France	0.67	8.0	Yes
LAP0111ab	Ares	Cultivar	Spring-type	France	0.73	8.5	Yes
Dieta	Dieta	Cultivar	Spring-type	UK	0.56	7.9	No
LAP0115ab	Energy	Cultivar	Spring-type	France	0.75	8.4	Yes
Feodora	Feodora	Cultivar	Spring-type	France	0.56	7.7	No
Frieda	Frieda	Cultivar	Spring-type	Germany	0.73	8.4	No
LAP0112ab	Lublanc	Cultivar	Spring-type	France	0.90	9.5	Yes
LAP0150abcd	Lucky	Cultivar	Spring-type	France	0.12	4.9	No

Arsenio	Arsenio	Cultivar	Mediterranean-type	Italy	0.29	6.4	No
LAP0156ab	Multitalia	Cultivar	Mediterranean-type	Italy	0.17	5.2	No
Murringo	Murringo	Cultivar	Mediterranean-type	Australia	0.58	7.5	No
Pecosa	Pecosa	Cultivar	Mediterranean-type	Chile	0.13	4.4	No
LAP0155a	Rumbo	Cultivar	Mediterranean-type	Chile	0.47	7.4	No
LAP0117ab	Adam	Cultivar	Winter-type	France	0.20	5.8	Yes
LAP0119ab	Aster	Cultivar	Winter-type	France	0.37	6.3	Yes
LAP0126ab	Ludet	Cultivar	Winter-type	France	0.09	4.8	Yes
LAP0118ab	Luxe	Cultivar	Winter-type	France	0.20	5.6	Yes

Table S2. List of 144 white lupin breeding lines (reference population 2). For each line, the following information is reported: name, cross, female parent, male parent, and mean values of plant mortality proportion and visual score of biomass injury assessed at −11°C freezing temperature in a phenotyping platform.

Line	Cross	Female parent	Male parent	Mortality	Visual score
1.09	1	Lucky	Gr56	0.52	7.4
1.22	1	Lucky	Gr56	0.45	7.5
1.31	1	Lucky	Gr56	0.59	8.1
1.44	1	Lucky	Gr56	0.50	7.3
1.51	1	Lucky	Gr56	0.53	7.5
1.62	1	Lucky	Gr56	0.35	6.9
1.71	1	Lucky	Gr56	0.25	6.6
1.74	1	Lucky	Gr56	0.30	6.4
1.87	1	Lucky	Gr56	0.49	7.3
2.08	2	MB-38	Gr56	0.17	6.3
2.29	2	MB-38	Gr56	0.28	5.9
2.47	2	MB-38	Gr56	0.39	7.5
2.56	2	MB-38	Gr56	0.30	7.0
2.70	2	MB-38	Gr56	0.29	6.2
2.91	2	MB-38	Gr56	0.44	7.4
3.03	3	Arsenio	Gr56	0.77	8.9
3.07	3	Arsenio	Gr56	0.61	8.0
3.09	3	Arsenio	Gr56	0.74	8.9
3.17	3	Arsenio	Gr56	0.50	7.6
3.35	3	Arsenio	Gr56	0.68	8.2
3.39	3	Arsenio	Gr56	0.66	8.2
3.42	3	Arsenio	Gr56	0.41	6.8
3.75	3	Arsenio	Gr56	0.85	9.4
4.05	4	L27PS3	Gr56	0.16	5.9
4.07	4	L27PS3	Gr56	0.31	7.0
4.12	4	L27PS3	Gr56	0.67	8.4
4.23	4	L27PS3	Gr56	0.78	8.6
4.25	4	L27PS3	Gr56	0.29	6.8
4.53	4	L27PS3	Gr56	0.68	8.5
4.56	4	L27PS3	Gr56	0.49	8.3
4.57	4	L27PS3	Gr56	0.30	7.3
4.66	4	L27PS3	Gr56	0.86	9.6
4.67	4	L27PS3	Gr56	0.50	7.7
5.06	5	Lucky	La646	0.73	8.6
5.12	5	Lucky	La646	0.51	7.9
5.23	5	Lucky	La646	0.50	7.4
5.28	5	Lucky	La646	0.61	8.1
5.34	5	Lucky	La646	0.65	8.5
5.40	5	Lucky	La646	0.50	7.5
5.43	5	Lucky	La646	0.37	7.1
5.50	5	Lucky	La646	0.52	7.5
5.51	5	Lucky	La646	0.70	8.6
6.01	6	MB-38	La646	0.40	7.3

6.30	6	MB-38	La646	0.21	6.3
6.41	6	MB-38	La646	0.80	8.8
6.43	6	MB-38	La646	0.25	6.2
6.45	6	MB-38	La646	0.05	5.7
6.47	6	MB-38	La646	0.09	6.3
6.58	6	MB-38	La646	0.68	8.3
6.62	6	MB-38	La646	0.24	6.6
6.65	6	MB-38	La646	0.16	6.1
6.71	6	MB-38	La646	0.60	8.2
7.10	7	Arsenio	La646	0.46	7.5
7.25	7	Arsenio	La646	0.57	8.2
7.26	7	Arsenio	La646	0.38	7.2
7.41	7	Arsenio	La646	0.77	8.7
7.46	7	Arsenio	La646	0.56	8.3
7.47	7	Arsenio	La646	0.77	9.2
7.54	7	Arsenio	La646	0.72	8.7
7.56	7	Arsenio	La646	0.62	8.0
7.72	7	Arsenio	La646	0.58	7.9
8.02	8	L27PS3	La646	0.69	8.7
8.08	8	L27PS3	La646	0.71	8.6
8.14	8	L27PS3	La646	0.73	8.7
8.18	8	L27PS3	La646	0.87	9.7
8.34	8	L27PS3	La646	0.74	8.7
8.37	8	L27PS3	La646	0.91	9.7
8.49	8	L27PS3	La646	0.66	8.6
8.51	8	L27PS3	La646	0.61	8.1
8.55	8	L27PS3	La646	0.76	9.0
9.08	9	Lucky	La246	0.78	8.8
9.16	9	Lucky	La246	0.66	9.1
9.42	9	Lucky	La246	0.43	7.2
9.54	9	Lucky	La246	0.60	8.5
9.58	9	Lucky	La246	0.55	8.0
9.60	9	Lucky	La246	0.67	8.3
9.78	9	Lucky	La246	0.44	8.1
9.81	9	Lucky	La246	0.51	7.6
9.86	9	Lucky	La246	0.71	8.9
10.03	10	MB-38	La246	0.55	8.2
10.18	10	MB-38	La246	0.40	7.0
10.21	10	MB-38	La246	0.65	8.1
10.28	10	MB-38	La246	0.41	7.6
10.39	10	MB-38	La246	0.67	8.1
10.40	10	MB-38	La246	0.65	7.9
10.44	10	MB-38	La246	0.66	8.2
10.59	10	MB-38	La246	0.50	7.3
10.62	10	MB-38	La246	0.61	7.9
10.91	10	MB-38	La246	0.49	7.6
11.01	11	Arsenio	La246	0.55	8.1

11.02	11	Arsenio	La246	0.76	8.8
11.09	11	Arsenio	La246	0.77	9.2
11.10	11	Arsenio	La246	0.68	8.6
11.22	11	Arsenio	La246	0.69	8.7
11.23	11	Arsenio	La246	0.84	9.5
11.25	11	Arsenio	La246	0.73	8.4
11.37	11	Arsenio	La246	0.67	8.6
11.40	11	Arsenio	La246	0.44	7.1
11.57	11	Arsenio	La246	0.42	7.7
12.09	12	L27PS3	La246	0.87	9.5
12.12	12	L27PS3	La246	0.76	9.0
12.15	12	L27PS3	La246	0.66	8.4
12.25	12	L27PS3	La246	1.0	10.3
12.34	12	L27PS3	La246	0.82	9.3
12.36	12	L27PS3	La246	0.97	9.9
12.37	12	L27PS3	La246	0.75	8.8
12.44	12	L27PS3	La246	0.90	9.2
12.50	12	L27PS3	La246	0.92	9.8
13.08	13	Lucky	LAP123	0.91	9.2
13.19	13	Lucky	LAP123	0.65	8.1
13.24	13	Lucky	LAP123	0.94	9.7
13.27	13	Lucky	LAP123	0.65	8.4
13.31	13	Lucky	LAP123	0.75	8.5
13.32	13	Lucky	LAP123	0.89	9.2
13.45	13	Lucky	LAP123	0.91	9.1
13.50	13	Lucky	LAP123	0.91	9.3
13.55	13	Lucky	LAP123	0.60	7.7
13.60	13	Lucky	LAP123	0.68	8.3
13.62	13	Lucky	LAP123	0.81	9.2
14.01	14	MB-38	LAP123	0.71	8.9
14.02	14	MB-38	LAP123	0.73	8.7
14.03	14	MB-38	LAP123	0.74	9.0
14.04	14	MB-38	LAP123	0.69	8.7
15.04	15	Arsenio	LAP123	0.65	8.0
15.05	15	Arsenio	LAP123	0.65	8.1
15.10	15	Arsenio	LAP123	0.59	8.1
15.17	15	Arsenio	LAP123	0.73	8.9
15.29	15	Arsenio	LAP123	0.76	8.7
15.32	15	Arsenio	LAP123	0.66	8.8
15.33	15	Arsenio	LAP123	0.67	8.9
15.36	15	Arsenio	LAP123	0.89	9.6
15.62	15	Arsenio	LAP123	0.89	9.4
15.91	15	Arsenio	LAP123	0.72	8.8
16.10	16	L27PS3	LAP123	0.85	9.3
16.20	16	L27PS3	LAP123	0.78	9.0
16.22	16	L27PS3	LAP123	0.83	9.3
16.29	16	L27PS3	LAP123	0.91	9.7

16.35	16	L27PS3	LAP123	0.94	9.4
16.37	16	L27PS3	LAP123	0.73	9.0
16.39	16	L27PS3	LAP123	0.96	9.4
16.52	16	L27PS3	LAP123	0.92	9.7
16.62	16	L27PS3	LAP123	0.74	8.8
16.77	16	L27PS3	LAP123	0.72	8.8
16.88	16	L27PS3	LAP123	0.81	9.3

Table S3. Extent of linkage disequilibrium (LD) of white lupin landrace and cultivar genotypes (reference population 1) and breeding lines (reference population 2). LD extent values were estimated as the physical distances at which the LD decay curves crossed the population-specific critical r^2 threshold (0.046 for reference population 1; 0.067 for reference population 2), above which LD is likely due to physical linkage.

Chromosome	Reference population	LD extent (base pairs)
1	1	4134
1	2	10256
2	1	3649
2	2	9724
3	1	4295
3	2	9158
4	1	3611
4	2	11965
5	1	3368
5	2	7615
6	1	2862
6	2	5565
7	1	2577
7	2	5443
8	1	3794
8	2	5520
9	1	3152
9	2	5724
10	1	3117
10	2	5617
11	1	3751
11	2	6152
12	1	4970
12	2	10846
13	1	4044
13	2	7849
14	1	2940
14	2	6639
15	1	2513
15	2	3703
16	1	3151
16	2	6289
17	1	4464
17	2	8663
18	1	4213
18	2	12365
19	1	5291
19	2	8262
20	1	2837

20	2	5676
21	1	7248
21	2	7756
22	1	3822
22	2	6489
23	1	3475
23	2	6405
24	1	2791
24	2	4658
25	1	3925
25	2	5018

Table S4. List of significant SNPs detected by a GWAS conducted on white lupin landrace and cultivar genotypes (reference population 1) and breeding lines (reference population 2) for plant mortality and a visual score of biomass injury assessed at −11°C freezing temperature in a phenotyping platform, according to the false discovery rate threshold at 5%. The negative logarithm of *p*.value (-log10(*p*)), minor allele frequency (MAF), and estimated trait effect are reported for each SNP.

**SNP**	**Population**	**Trait**	**$-\log_{10}(p)$**	**MAF**	**Effect**
Chr05_4820341	1	Mortality	7.33	0.11	-0.07
Chr06_1878682	1	Mortality	7.88	0.19	0.06
Chr08_3511620	1	Mortality	5.49	0.31	0.04
Chr13_15386976	1	Mortality	11.75	0.33	-0.06
Chr14_10127694	1	Mortality	6.81	0.11	0.07
Chr16_5032297	1	Visual score of biomass injury	6.58	0.06	-0.61
Chr23_1146188	1	Mortality	11.59	0.15	-0.08
Chr23_1146188	1	Visual score of biomass injury	9.13	0.15	-0.48
Chr02_14306413	2	Mortality	9.05	0.06	-0.12
Chr02_14306413	2	Visual score of biomass injury	12.89	0.06	-0.73
Chr04_7632627	2	Mortality	7.31	0.29	0.06
Chr19_17886074	2	Mortality	6.55	0.10	-0.06
Chr21_1050253	2	Mortality	7.44	0.25	0.06

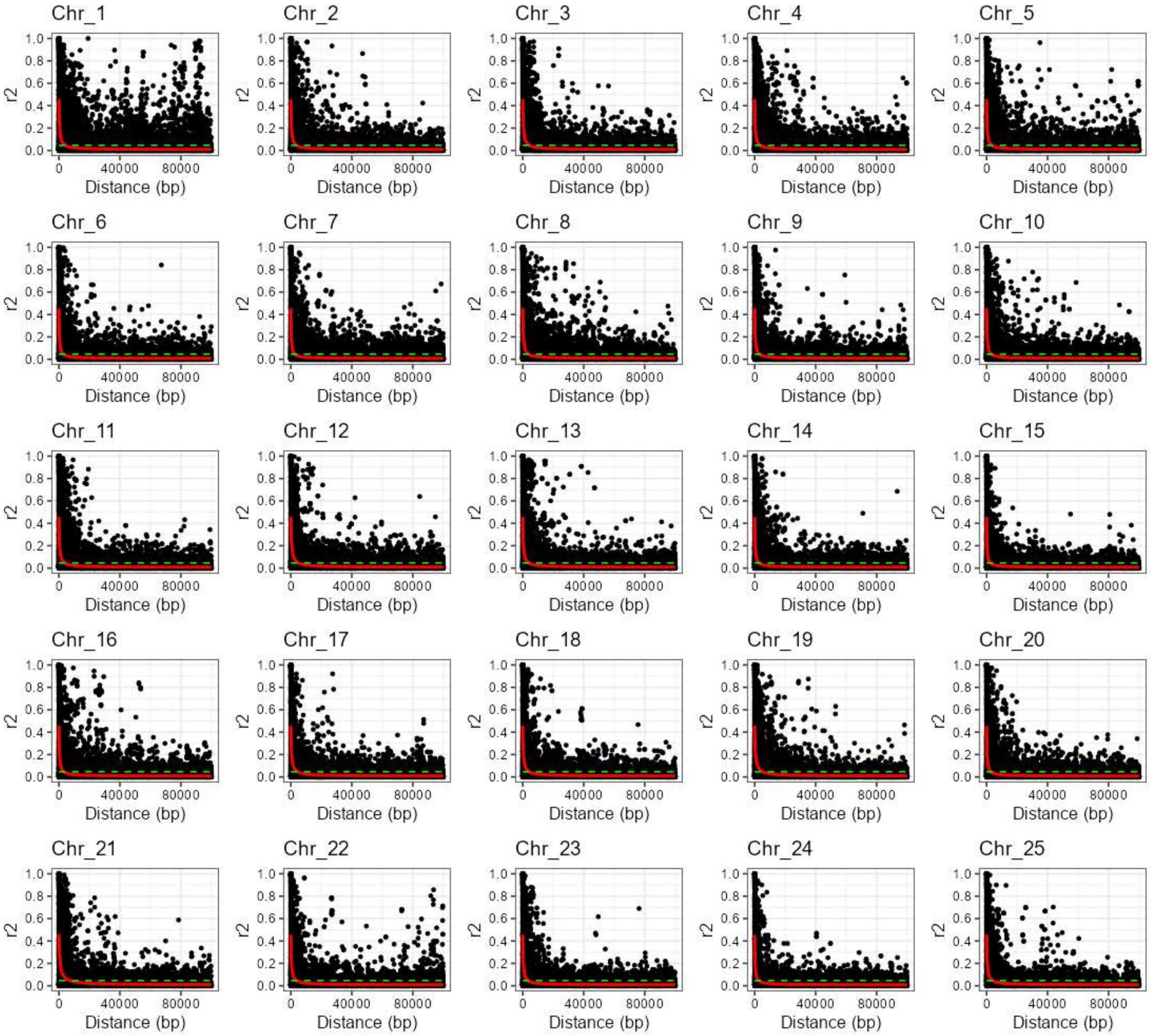

Figure S1. LD decay plots for each chromosome for white lupin landrace and cultivar genotypes (reference population 1), based on r^2 values (Y axis) and physical distance in base pairs (X axis), estimated on pairwise combinations of 40,914 SNPs within a 100 kb window.

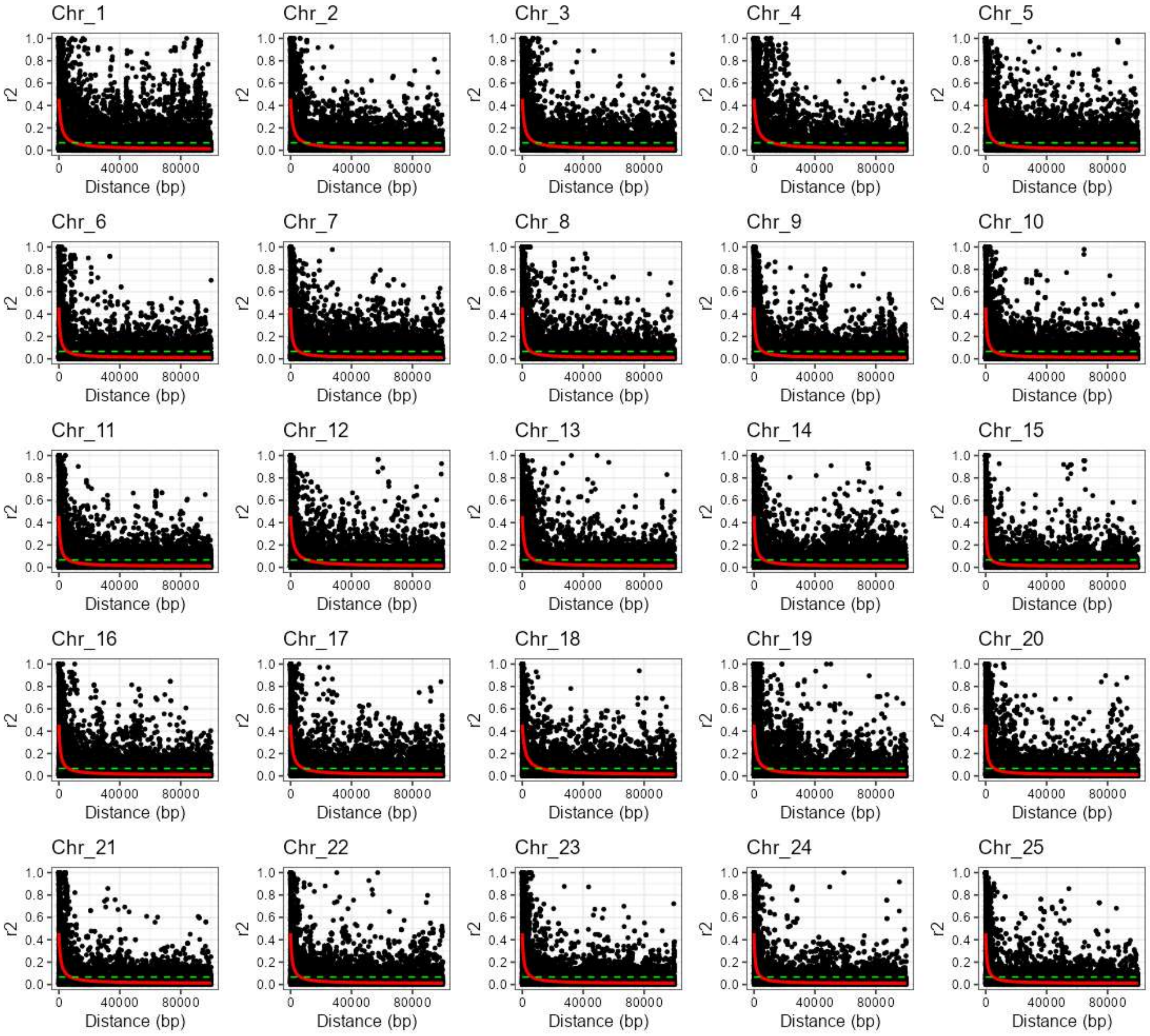

Figure S2. LD decay plots for chromosome for white lupin breeding lines (reference population 2) based on r^2 values (Y axis) and physical distance in base pairs (X axis), estimated on pairwise combinations of 32,951 SNPs within a 100 kb window.

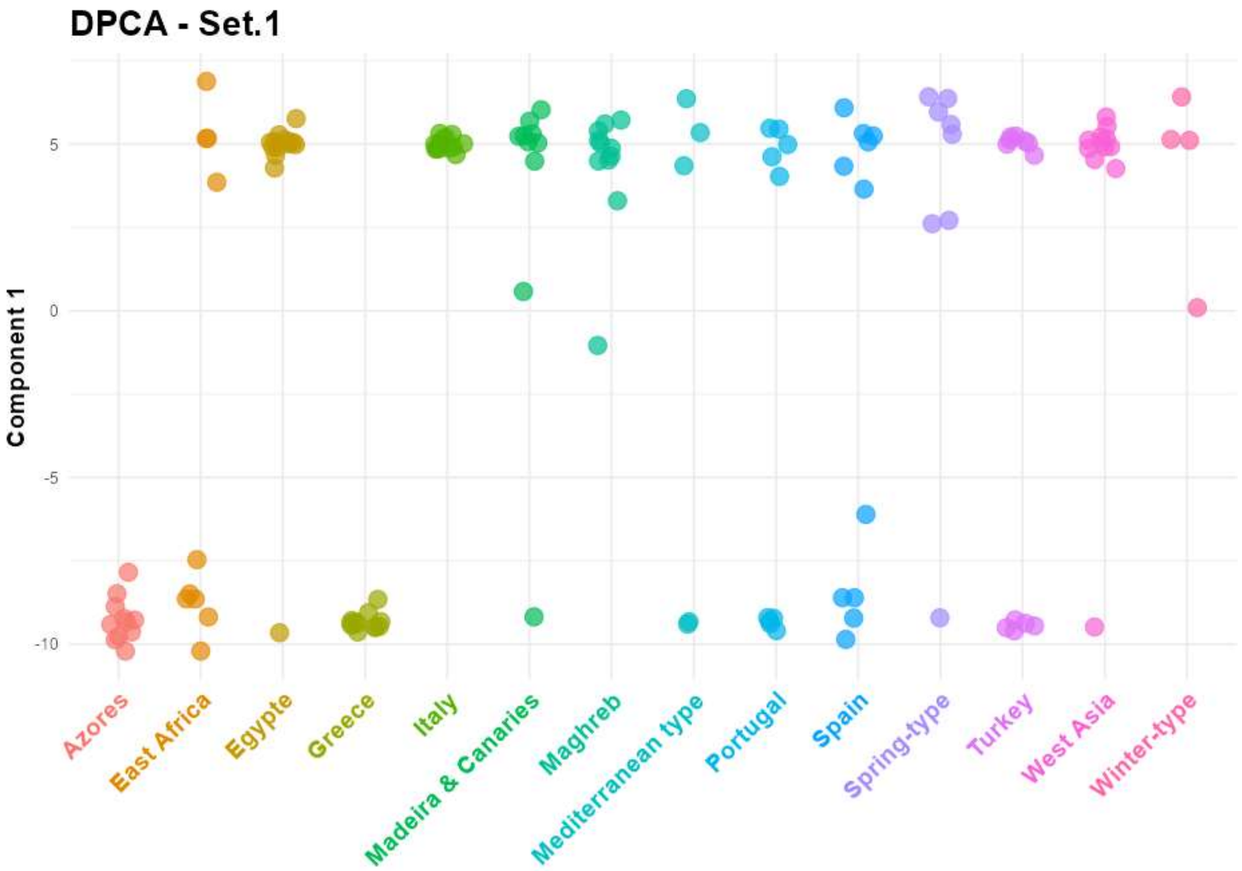

Figure S3. Population structure of landrace and cultivar genotypes (reference population 1) based on the first discriminant function (component) of a discriminant principal component analysis. Genotypes are grouped into 14 gene pools based on origin (landraces) or phenological class (cultivars).

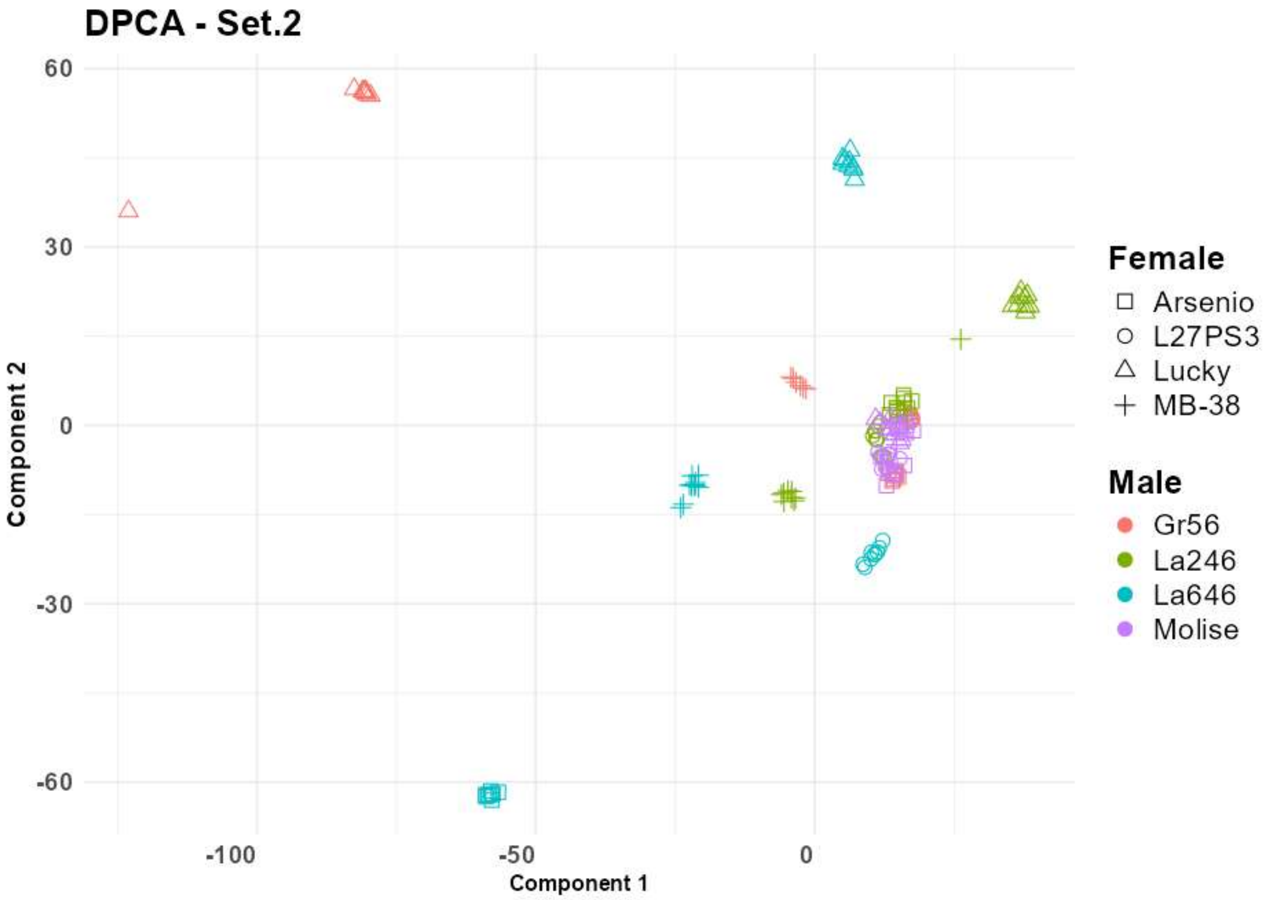

Figure S4. Population structure of the breeding lines (reference population 2), based on the first two discriminant functions (components) of a discriminant principal component analysis. The symbol shapes represent the sweet-seed parent line (female), while the symbol colors represent the bitter-seed parent landrace.

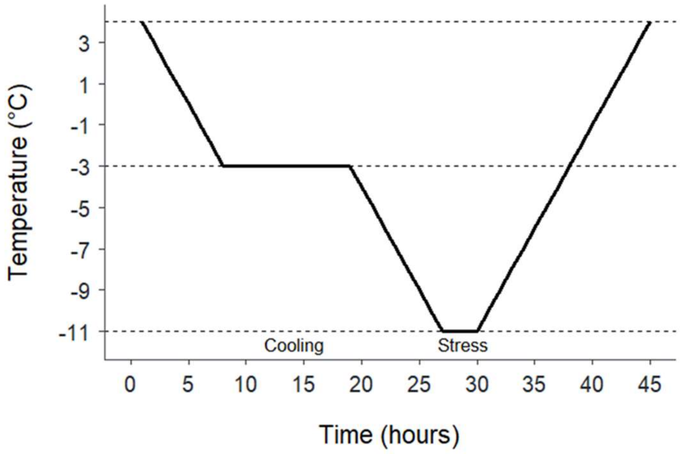

Figure S5. Temperature profile showing the decrease and subsequent increase during the cooling and frost treatment.

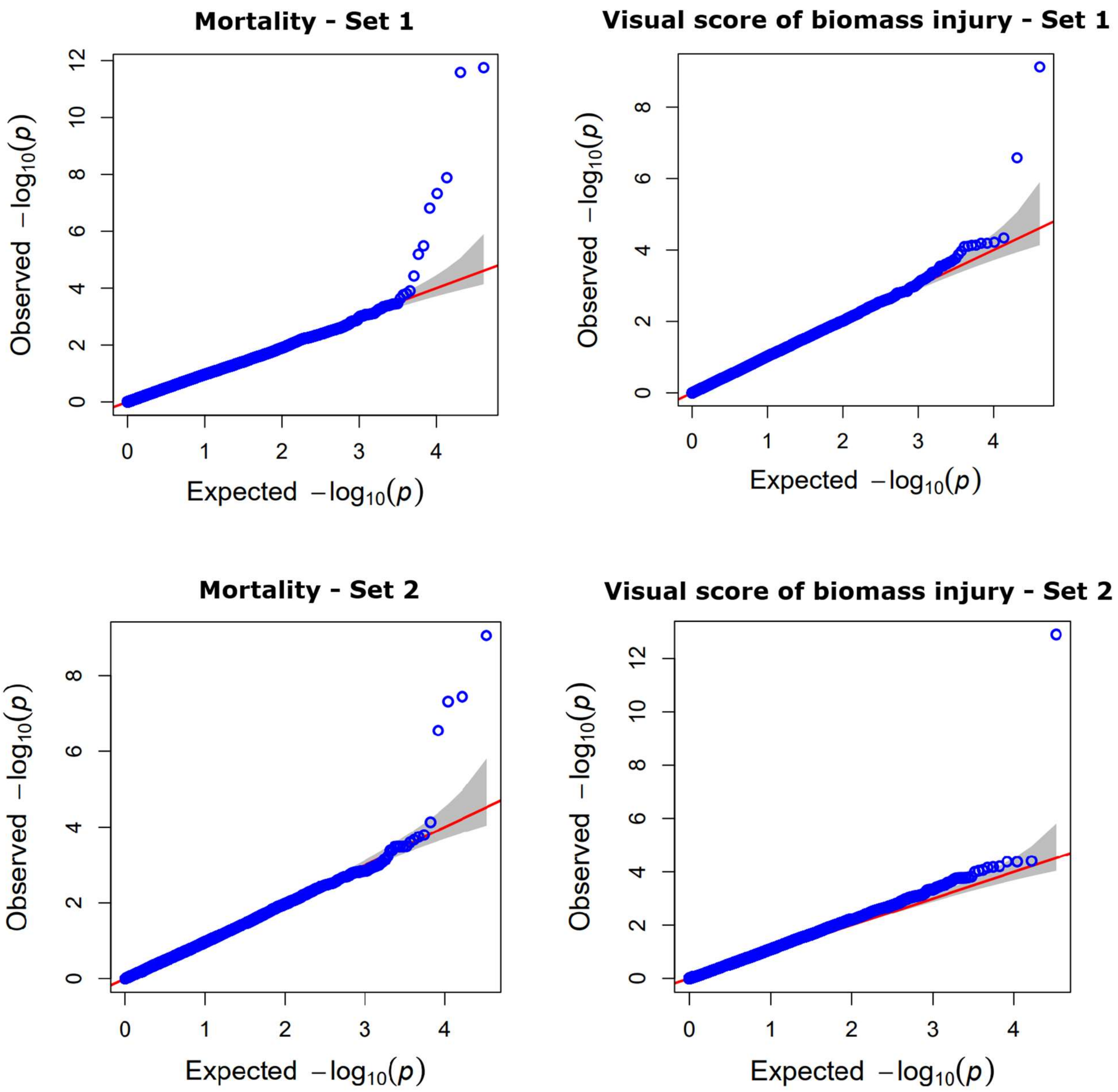

Figure S6. Quantile-Quantile plots of expected vs. observed association scores for (a) 40,914 SNPs from 144 landrace and cultivar genotypes (reference population 1), and (b) 32,951 SNPs of 144 breeding lines (reference population 2), with plant mortality and visual score of biomass injury assessed at −11°C freezing temperature in a phenotyping platform. The red line indicates equality between expected and observed quantiles; the grey area represents the associated 95% confidence interval.
